# Discovery of ITI-333, a Novel Orally Bioavailable Molecule Targeting Multiple Receptors for the Treatment of Pain and Other Disorders

**DOI:** 10.1021/acs.jmedchem.4c00480

**Published:** 2024-05-28

**Authors:** Peng Li, Qiang Zhang, Hailin Zheng, Yupu Qiao, Gretchen L. Snyder, Terry Martin, Wei Yao, Lei Zhang, Robert E. Davis

**Affiliations:** Intra-Cellular Therapies, Inc., 430 East 29th Street, Suite 900, New York, New York 10016, United States

## Abstract

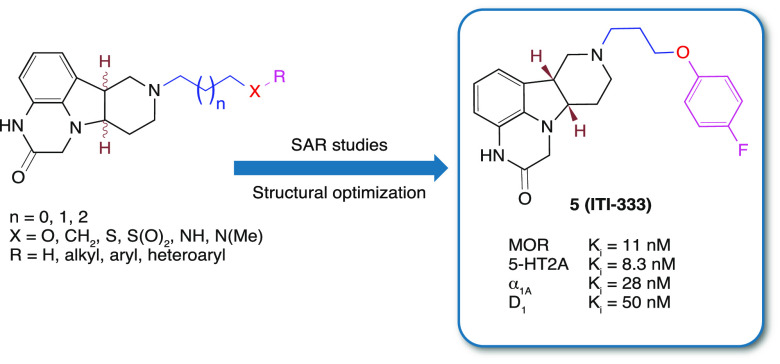

Development of more efficacious medications with improved safety profiles to manage and treat multiple forms of pain is a critical element of healthcare. To this end, we have designed and synthesized a novel class of tetracyclic pyridopyrroloquinoxalinone derivatives with analgesic properties. The receptor binding profiles and analgesic properties of these tetracyclic compounds were studied. Systematic optimizations of this novel scaffold culminated in the discovery of the clinical candidate, (6*bR*,10*aS*)-8-[3-(4-fluorophenoxy)propyl]-6*b*,7,8,9,10,10*a*-hexahydro-1*H*-pyrido[3′,4′:4,5]pyrrolo[1,2,3-*de*]quinoxalin-2(3*H*)-one (compound **5**, ITI-333), which exhibited potent binding affinity to serotonin 5-HT_2A_ (*K*_*i*_ = 8.3 nM) and μ-opioid receptors (MOR, *K*_*i*_ = 11 nM) and moderate affinity to adrenergic α_1A_ (*K*_*i*_ = 28 nM) and dopamine D_1_ (*K*_*i*_ = 50 nM) receptors. ITI-333 acts as a 5-HT_2A_ receptor antagonist, a MOR partial agonist, and an adrenergic α_1A_ receptor antagonist. ITI-333 exhibited dose-dependent analgesic effects in rodent models of acute pain. Currently, this investigational new drug is in phase I clinical development.

## Introduction

**Figure 1:**
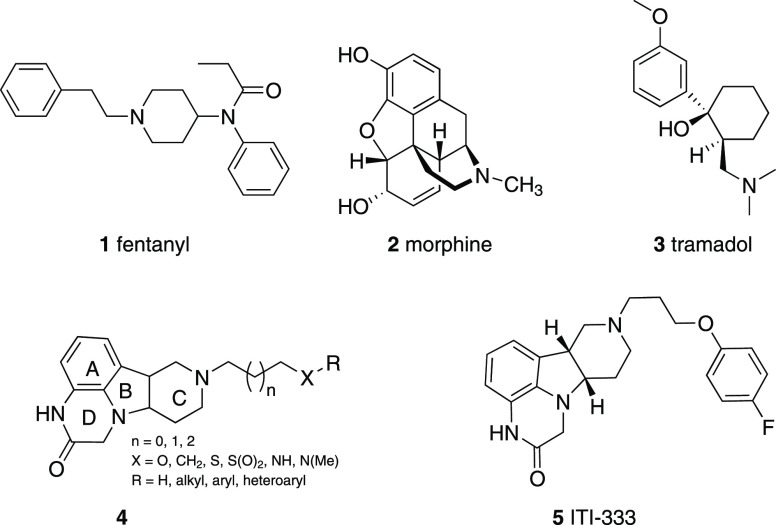
Structures of fentanyl (**1**), morphine (**2**), tramadol (**3**), polycyclic scaffold (**4**), and ITI-333 (**5**).

Pain in its many forms is debilitating and affects a significant portion of the U.S. population. It is estimated that up to 50 million adults in the United States experience some form of pain, resulting in substantial healthcare costs and lost productivity.^1^ The total economic and societal cost of pain is estimated to be at least six hundred billion dollars.^2^ While a variety of prescribed or over-the-counter medications are available for the treatment of various forms of pain, opioid analgesics, especially those acting at the μ-opioid receptor (MOR), are proven effective, such as fentanyl (**1**, Figure 1), morphine (**2**), and tramadol (**3**). However, the use of MOR-targeting opioids is often associated with abuse liability and serious adverse effects, including tolerance, respiratory depression, sedation, and constipation, which limit their utility and present risks to society when misused.^3−5^

To mitigate the adverse reactions associated with conventional MOR-targeting drugs and develop more effective medications for the treatment of pain, various new approaches have been attempted.^6−9^ Developing MOR-positive allosteric modulators could be an attractive approach. Modulating the activity of the MOR only when an orthosteric opioid receptor agonist occupies the receptor could maintain spatial and temporal control of receptor signaling in vivo while potentially leading to fewer tolerance and dependence issues.^10,11^ Non-MOR strategies have also been explored to treat opioid-induced respiratory depression, including α-amino-3-hydroxy-5-methyl-4-isoxazolepropionate receptor positive allosteric modulators^12,13^ and serotonin 5-HT_1A_ receptor agonists.^14,15^ α_2_-Adrenergic receptor agonists can also be used to alleviate symptoms of opioid withdrawal by dampening the release of norepinephrine, which has effects on autonomic nervous system control over some symptoms associated with withdrawal. For instance, lofexidine has recently been approved for mitigation of opioid withdrawal symptoms to facilitate abrupt opioid discontinuation in adults.^16,17^ Serotonin 5-HT_2C_ agonists and selective serotonin reuptake inhibitors (SSRI) were also found to be effective at suppressing opioid craving and alleviating withdrawal symptoms.^18,19^ It is worth noting that bifunctional or multifunctional ligands—targeting multiple receptors in addition to opioid receptors—could potentially deliver better efficacy for the treatment of pain and opioid use disorder with fewer adverse effects.^20−22^

During the development of drug candidates for the treatment of neurological and psychiatric disorders, we have built a large, diversified CNS-active compound library. Many compounds in this library are serotonin 5-HT_2A_ antagonists. Serotonin 5-HT_2A_ receptors have been extensively studied over the years and found to play an important role in the treatment of psychosis, depression, anxiety, pain, and possibly alcohol and cocaine dependence.^23,24^ Anxiety and depression, key symptoms of the cocaine withdrawal syndrome in human addicts, are considered the main factors that precipitate relapse in chronic cocaine addiction. Mirtazapine, a 5-HT_2A_ receptor antagonist, has been shown to attenuate anxiety- and depression-like behaviors in rats during cocaine withdrawal.^25^ Blockade of serotonin 5-HT_2A_ receptors, which suppresses behavioral sensitization and naloxone-precipitated withdrawal symptoms in morphine-treated mice,^26^ also suppresses cue-evoked reinstatement of cocaine-seeking behavior in a rat self-administration model.^27^ In addition, on a cellular level, MOR and 5-HT_2A_ receptors were found to be colocalized in several distinct regions of rat brain, including the periaqueductal gray, which is associated with opioid-induced analgesia and tolerance.^28^ Combination use of a 5-HT_2A_ antagonist with a MOR partial agonist, or developing novel molecules targeting both receptors, could be an attractive approach for the treatment of pain with improved efficacy and reduced adverse effects. By screening the in-house compound library, we identified a novel class of compounds that bind both 5-HT_2A_ and MOR, as shown in Figure 1.^29^ Herein, we report the synthesis, structure–activity relationships (SAR), pharmacokinetic properties, and efficacy of these polycyclic compounds (**4**, Figure 1), as well as the discovery and evaluation of the development candidate, ITI-333. Currently, clinical studies of ITI-333 in humans are ongoing.

## Results and Discussion

### Chemistry

**Scheme 1:**
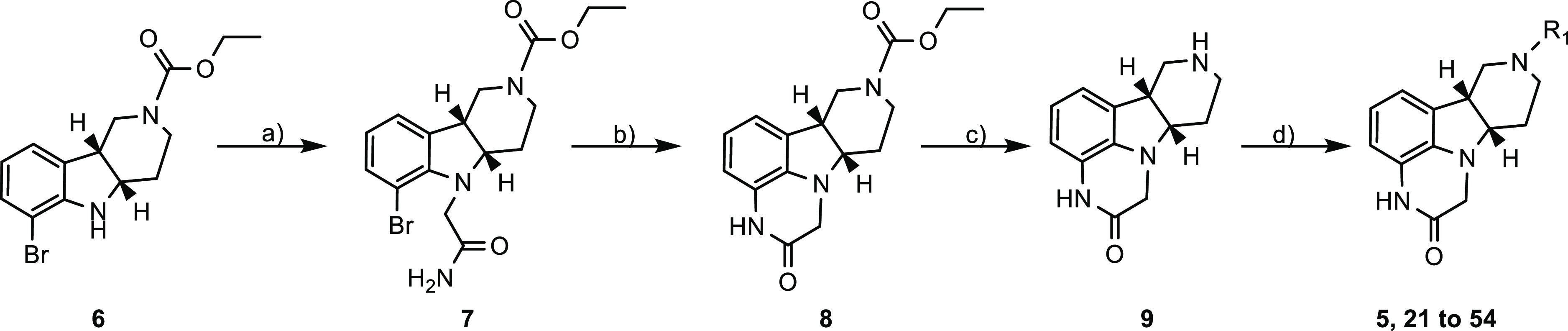
Synthesis of Pyridopyrroloquinoxalinone Derivatives with Different Side Chains Reagents and conditions: (a) chloroacetamide, KI, dioxane, 104 °C, 75% yield; (b) KI, CuI, dioxane, reflux, 79% yield; (c) 33% HBr in acetic acid, 50 °C, >99% yield; (d) R_1_X, DIPEA, DMF, 78 °C, 3–66% yield.

To efficiently synthesize a diverse set of tetracyclic pyridopyrroloquinoxalinone derivatives with various side chains for SAR studies, a general synthetic route has been developed, as illustrated in Scheme 1. Commercially available compound **6** was reacted with 2-chloroacetamide to give compound **7**. Intramolecular N-arylation of compound **7**, catalyzed by copper(I) iodide, provided intermediate **8**. After the removal of the ethoxycarbonyl protective group in intermediate **8** with hydrobromic acid (HBr) in acetic acid, the resulting intermediate **9** was alkylated with various alkyl halides to afford the final compounds **5**, **21**–**54** as listed in Tables 1–6.

**Table 1:**
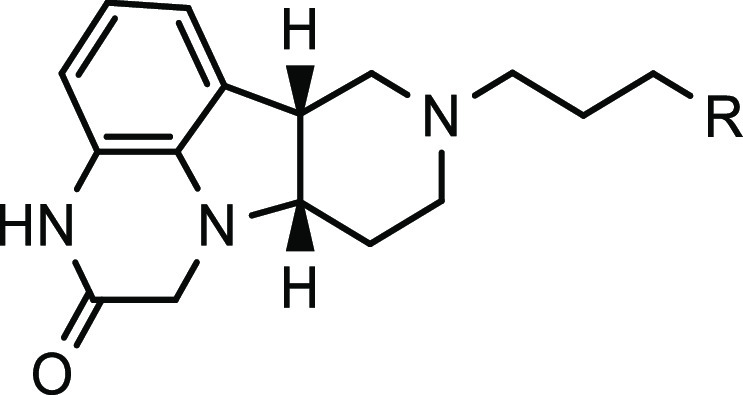

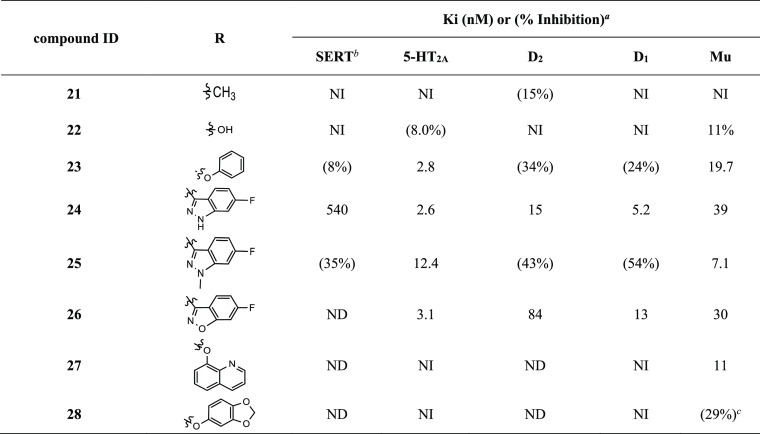
SAR of Compounds with Variations of Side Chains

a Inhibition at 100 nM.

b Inhibition at 200 nM.

c Inhibition at 30 nM. NI, no inhibition. ND, not determined. All reported potency values are the average of at least two independent measurements.

**Table 2:**
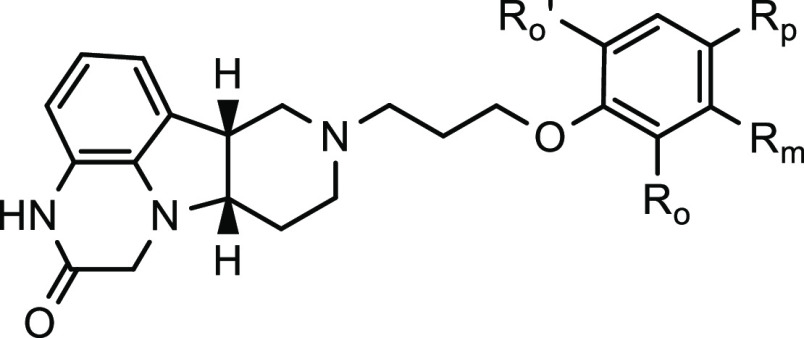
SAR of Compounds with Variations of Substituents on the Phenyl Ring

					*K*_*i*_ (nM) (% inhibition)^a^				
compound ID	*R*_p_	*R*_m_	*R*_o_	*R*_o_′	SERT	5-HT_2A_	D_2_	D_1_	Mu
**23**	H	H	H	H	(8.0%)^d^	2.8	(34%)	(24%)	19.7
						(84%)			(61%)
**5**	F	H	H	H	590	8.3	160	50	11
					(15%)^d^	(98%)	(26%)	(44%)	(86%)
**29**	Cl	H	H	H	ND	(15%)^b^	ND	NI	7.3
**30**	Br	H	H	H	(44%)	(70%)	(8.4%)	NI	(90%)
**31**	CN	H	H	H	ND	NI	ND	(7%)^c^	15
**32**	Me	H	H	H	(9.6%)	(62%)	(20%)	(13%)	(53%)
**33**	OH	H	H	H	(11%)	(77%)	(41%)	NI	(13%)
**34**	MeO	H	H	H	(10%)	NI	ND	NI	NI
**35**	OBn	H	H	H	(68%)	(76%)	(23%)	(4.6%)	(56%)
**36**	H	H	F	H	(5.1%)	(87%)	(28%)	(18%)	(33%)
**37**	H	F	H	H	(7.7%)	(78%)	(14%)	(32%)	(72%)
**38**	F	H	H_2_NCO	H	(9.6%)	(14%)	NI	NI	(76%)
**39**	F	F	H	H	508	16.1	1620	232	1.8
					(15%)^d^	(71%)	(7%)	(15%)	(91%)
**40**	F	H	F	H	NI	15.2	(36%)	(21%)	(61%)
						(85%)			
**41**	F	OH	H	H	(13%)	110	NI	(25%)	(23%)
						(46%)			
**42**	F	H	OH	H	(5%)	19	67	(22%)	(22%)
						(70%)	(45%)		
**43**	F	H	Me	Me	(34%)^d^	(40%)	NI	NI	4.3
									(84%)

a Inhibition at 100 nM.

b Inhibition at 10 nM.

c Inhibition at 50 nM.

d Inhibition at 200 nM. NI, no inhibition. ND, not determined. All reported potency values are the average of at least two independent measurements.

**Table 3:**
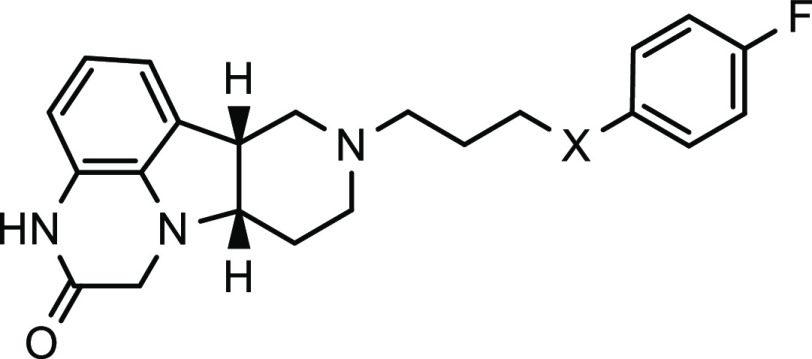
SAR of Compounds with Variations of Heteroatoms of the Side Chain

		*K*_*i*_ (nM) (% inhibition)^a^					
compound ID	X	SERT^b^	5-HT_2A_	D_2_	D_1_	Mu	LLE^c^
**5**	O	590 (15%)	8.3 (98%)	160 (26%)	50 (44%)	11 (86%)	4.31
**44**	CH_2_	(16%)	3.7 (85%)	(24%)	(32%)	4.1 (88%)	4.19
**45**	S	NI	2.5 (93%)	(49%)	(54%)	12.9 (73%)	3.78
**46**	S(O)_2_	NI	(33%)	NI	NI	NI	
**47**	NH	NI	(32%)	(25%)	(11%)	10.8 (83%)	4.51
**48**	N (Me)	(53%)	(76%)	(14%)	(21%)	(22%)	

a Inhibition at 100 nM.

b Inhibition at 200 nM.

c LLE is ligand–lipophilicity efficiency calculated based upon compound ClogP and binding affinity to MOR. NI, no inhibition. ND, not determined. All reported potency values are the average of at least two independent measurements.

**Table 4:**
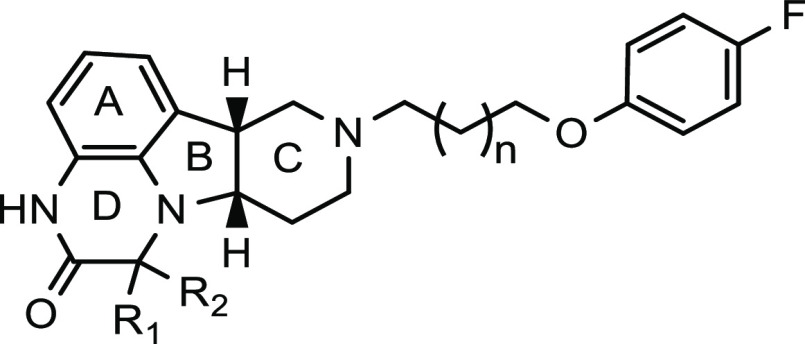
SAR of Compounds with Different Lengths of Side Chains and Variations of Substituents on the D-Ring

				*K*_*i*_ (nM) or % inhibition^a^				
compound ID	*n*	R_1_	R_2_	SERT	5-HT_2A_	D_2_	D_1_	Mu
**49**	0	H	H	NI	(48%)	(24%)	(10%)	(30%)
**5**	1	H	H	590 (15%)^b^	8.3 (98%)	160 (26%)	50 (44%)	11 (86%)
**50**	2	H	H	NI	(37%)	(27%)	(5.4%)	(39%)
**51**	1	H	Me	NI	(88%)	(49%)	(26%)	(40%)
**52**	1	Me	Me	NI	8.6 (89%)	53 (57%)	(27%)	(8%)

a Inhibition at 100 nM.

b Inhibition at 200 nM. NI, no inhibition. ND, not determined. All reported potency values are the average of at least two independent measurements.

**Table 5:**
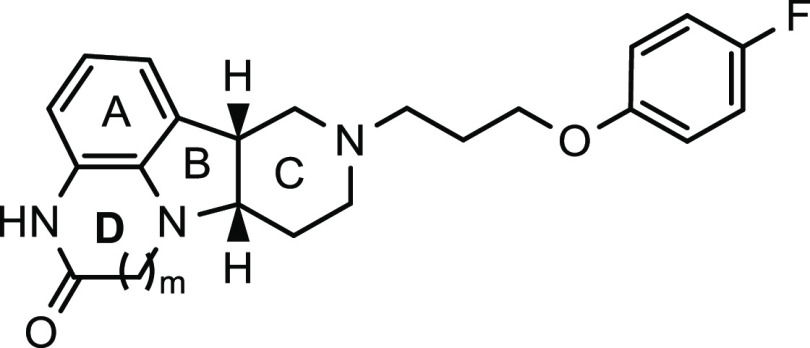
SAR of Compounds with Variations of the D-Ring

		*K*_*i*_ (nM) or % inhibition^a^				
compound ID	*m*	SERT^b^	5-HT_2A_	D_2_	D_1_	Mu
**14**	0	NI	(90%)	(37%)	(37%)	(24%)
**5**	1	590 (15%)	8.3 (98%)	160 (26%)	50 (44%)	11 (86%)
**53**	2	(29%)	(93%)	(24%)	(14%)	(38%)

a Inhibition at 100 nM.

b Inhibition at 200 nM. NI, no inhibition. ND, not determined. All reported potency values are the average of at least two independent measurements.

**Table 6:**
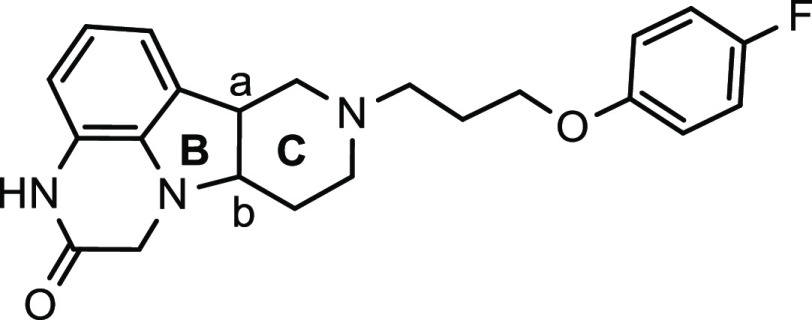
SAR of Compounds with Different Chirality

			*K*_*i*_ (nM) or % inhibition^a^				
compound ID	*a*	*b*	SERT	5-HT_2A_	D_2_	D_1_	Mu
**5**	*R*	*S*	590 (15%)^b^	8.3 (98%)	160 (26%)	50 (44%)	11 (86%)
**54**	*S*	*R*	NI	11 (84%)	210 (18%)	75 (50%)	290 (9.6%)
**20**	racemic (*R*,*R* or *S*,*S*)		NI	>10,000	160 (27%)	5.6 (79%)	26 (64%)

a Inhibition at 100 nM.

b Inhibition at 200 nM. NI, no inhibition. ND, not determined. All reported potency values are the average of at least two independent measurements.

**Scheme 2:**
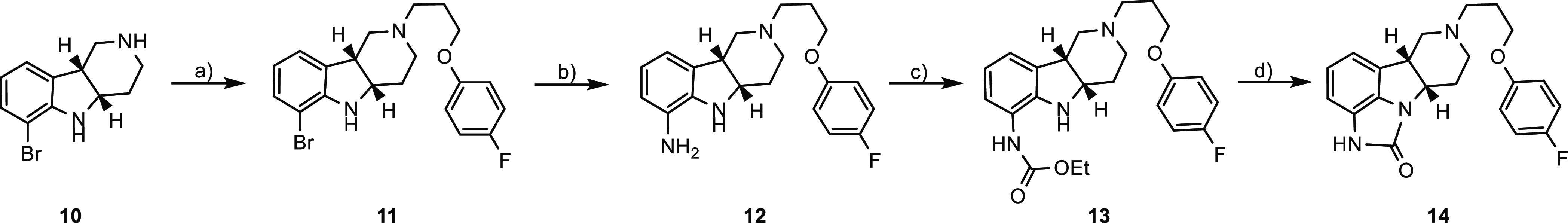
Synthesis of Compound **14** Reagents and conditions: (a) 1-(3-chloropropoxy)-4-fluorobenzene, potassium iodide, DIPEA, DMF, 95% yield. (b) Ammonium hydroxide solution (28.0–30.0% NH_3_ basis), copper iodide, 2,2,6,6-tetramethyl-3,5-heptanedione, cesium carbonate, DMAc, 62% yield. (c) Ethyl chloroformate, DMAP, 99% yield; (d) 1 M lithium bis(trimethylsilyl)amide in toluene, 51% yield.

To explore the SAR of different polycyclic ring systems, the second synthetic route was developed, as shown in Scheme 2. Compound **10** was N-alkylated with 1-(3-chloropropoxy)-4-fluorobenzene to give intermediate **11**. Amination of compound **11** with ammonium hydroxide solution resulted in compound **12**. Acylation of compound **12** with acyl chloride gave compound **13**, which was further cyclized to generate compound **14**.

**Scheme 3:**
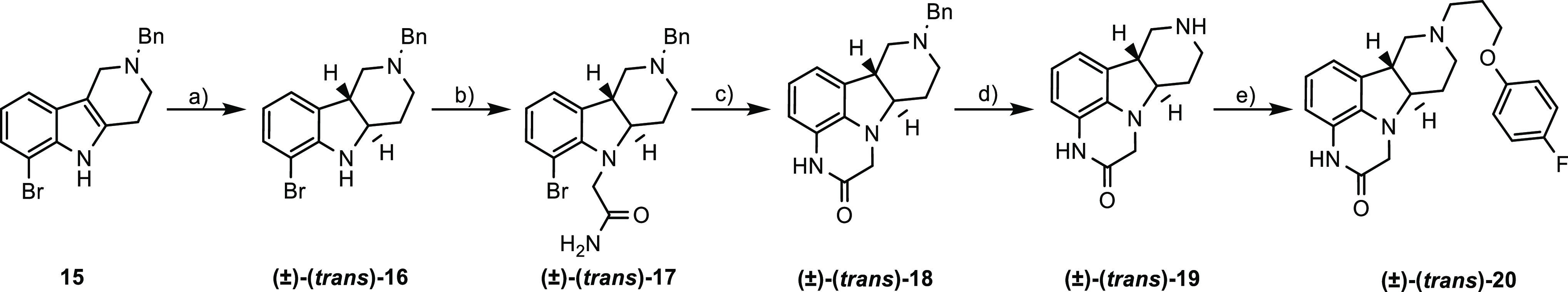
Synthesis of Compound **20** Reagents and conditions: (a) borane, 40 °C, 28% yield. (b) Lithium bis(trimethylsilyl)amide (1.0 M in toluene), chloroacetamide, 69% yield. (c) K_2_CO_3_, CuI, *N*,*N*,*N*′,*N*′-tetramethylethylenediamine, 100 °C, 21% yield. (d) Pd/C, H_2_, 57% yield. (e) 1-(3-Chloropropoxy)-4-fluorobenzene, potassium iodide, DIPEA, DMF, 10% yield.

As part of our SAR studies, racemic compound **20** with *trans*-stereochemistry of the fused ring system was also synthesized via the synthetic route described in Scheme 3. Compound **15** was reduced with borane to give intermediate racemic *trans*-**16**, which was subsequently reacted with 2-chloroacetamide to generate racemic compound *trans*-**17**. Cyclization of compound *trans*-**17**, followed by deprotection of the benzyl group produced intermediate racemic *trans*-**19**, which was alkylated with 1-(3-chloropropoxy)-4-fluorobenzene to afford the final racemic *trans* compound **20**.

### In Vitro Activities and SAR

The serotonin 5-HT_2A_ receptor binding affinities of polycyclic pyridopyrroloquinoxalinone derivatives were determined by radioligand displacement methods using human full-length recombinant serotonin 5-HT_2A_ receptors expressed in HEK-293E cells. (±)-1-(2,5-Dimethoxy-4-[^125^I]-iodophenyl)-2-aminopropane (^125^DOI) was used as a radioligand. The binding affinities of compounds against human recombinant dopamine D_2_ short receptor, expressed in HEK-293 cells, were determined with [^3^H]7-OH-DPAT as a radioligand. The binding affinities of compounds against human recombinant dopamine D_1_ receptor, expressed in Chinese hamster ovary (CHO) cells, were determined with [^3^H]SCH 23390 as a radioligand. The binding affinities of compounds against human recombinant MOR, expressed in HEK-293 cells, were determined with [^3^H]DAMGO as a radioligand. The binding affinities of compounds against human recombinant 5-HT transporter (SERT), expressed in CHO cells, were determined with [^3^H]imipramine as a radioligand.

To delineate the SAR around the investigational new drug **5** (ITI-333), five representative sets of compounds were evaluated as summarized in Tables 1–5. In addition to the potential of binding to MOR and serotonin 5-HT_2A_ receptors, this series of tetracyclic compounds could potentially bind to dopamine D_1_ and D_2_ receptors and SERT, depending on the structural features of the polycyclic cores and linked side chains. Thus, screening assays against these five targets were conducted to explore the SAR around this polycyclic novel scaffold. Initially, the SAR of tetracyclic pyridopyrroloquinoxalinone derivatives with various side chains is summarized in Table 1. When a butyl group was linked to the tetracyclic core, the resulting compound **21** did not exhibit any meaningful binding affinity to serotonin 5-HT_2A_, dopamine D_1_ and D_2_, μ-opioid receptors, or the SERT. Similarly, the hydroxypropyl-substituted analogue **22** is inactive against these targets. It is remarkable that replacing the terminal hydroxy group on the side chain of **22** with a phenoxy group led to compound **23** with potent binding affinity to MOR (*K*_*i*_ = 19.7 nM) and serotonin 5-HT_2A_ receptors (*K*_*i*_ = 2.8 nM). To further explore this finding, we focused our SAR study on the analogues with various aromatic or heterocyclic ring systems on the side chains. As shown in Table 1, when the phenoxy group on the side chain is replaced with a 6-fluoroindazolyl group, the resulting analogue **24** maintained binding affinity to 5-HT_2A_ receptors (*K*_*i*_ = 2.6 nM) and showed slightly weaker affinity to MOR (*K*_*i*_ = 39 nM) relative to compound **23**. Interestingly, the bigger planar ring system on the side chain offered a compound with potent binding affinity to dopamine D_1_ (*K*_*i*_ = 5.2 nM) and D_2_ (*K*_*i*_ = 15 nM) receptors, as well as moderate inhibition of SERT (*K*_*i*_ = 540 nM). N-methylation of the indazole ring led to weakened affinity of compound **25**, compared to compound **24**, to 5-HT_2A_, D_1_, and D_2_ receptors, while the binding affinity to MOR improved approximately 5-fold. Replacing the indazole component with a benzisoxazole ring system did not significantly alter receptor binding profiles except that the resulting compound **26** exhibited over 5-fold weaker affinity to dopamine D_2_ receptors relative to compound **24**. We also attempted to fuse the phenyl group on the side chain of compound **23** with another ring system to explore the SAR. Remarkably, compound **27**, with a pyridyl ring fused with the phenyl ring, became a selective ligand that binds to MOR only with a *K*_*i*_ of 11 nM. When a nonaromatic ring was fused with the phenyl ring, the resulting compound **28** showed a much weaker binding affinity to MOR. These findings suggested that receptor binding profiles of this class of compounds can be modulated by adjusting the aromatic ring systems on the side chains. While the MOR-selective ligand **27** could be an interesting lead candidate for further optimization, our interest lies in the development of drug-like candidates interacting with both serotonin 5-HT_2A_ and μ-opioid receptors, so compound **23** was selected for further optimization.

After the promising candidate **23** was discovered, the initial optimization of this compound involved modification of the para-position of the phenoxy group on the side chain, which is generally considered as a metabolically labile position. Various electron-withdrawing and electron-donating substituents with different sizes were introduced to this position, as shown in Table 2. Among the analogues with halogen groups substituted at the para-position of the phenyl ring, compound **5** (ITI-333) has the most potent and balanced affinity to both MOR (*K*_*i*_ = 11 nM) and serotonin 5-HT_2A_ receptors (*K*_*i*_ = 8.3 nM). In addition, capping this position with a fluoro group should offer improved metabolic stability relative to a chloro or bromo group. Compound **5** also showed moderate affinity to dopamine D_1_ (*K*_*i*_ = 50 nm) and D_2_ (*K*_*i*_ = 160 nM) receptors, as well as weak SERT inhibition (*K*_*i*_ = 590 nM). In contrast, compounds **29** and **30** do not bind to dopamine D_1_ receptors. When a cyano group was introduced, the resulting compound **31** maintained binding affinity to MOR (*K*_*i*_ = 15 nM), while its binding to 5-HT_2A_ receptors was abolished. These data indicated that a small electron-withdrawing group, such as a fluoro group at the para-position, is optimal for balanced high-affinity binding to MOR and 5-HT_2A_ receptors. When an electron-donating group was introduced at this position, weaker binding affinity to these receptors was observed. As shown in Table 2, Compounds **32** and **33** exhibited significantly weaker binding activity to the tested receptors, and the methoxy-substituted compound **34** only exhibited very weak inhibition to SERT and no engagement of other receptors. Interestingly, compound **35**, containing a bulky benzyloxy substituent at the para-position, regained some affinity to these receptors, compared to compound **34**. It can be hypothesized that the additional phenyl ring on the side chain created additional hydrophobic or π–π interactions with these receptors.

After the fluoro-substituted compound **5** was identified, we evaluated analogues with substituents at various positions of the phenyl ring on the side chain to further explore the SAR. Shifting the fluoro group from the para-position to the ortho- or meta-position led to weaker affinity to these receptors, as reflected by compounds **36** and **37** in Table 2. When two fluoro groups were introduced at the para- and ortho-positions of the phenyl ring, the resulting compound **40** did not show any improvement in binding affinity to the studied receptors. Remarkably, compound **39** with difluoro substitution at para- and meta-positions exhibited increased affinity to MOR (*K*_*i*_ = 1.8 nM), which is about 11-fold more potent than the unsubstituted parent compound **23**, while its affinity to 5-HT_2A_ receptors was reduced by approximately 6-fold relative to **23**. Changing the *meta*-fluoro group to a hydroxy group significantly decreased compound binding affinity to MOR and 5-HT_2A_ receptors (**39** vs **41**). Replacing the *ortho*-fluoro group with a hydrophilic amide group did not alter MOR binding affinity dramatically, while binding affinity to 5-HT_2A_ receptors was increased (**38** vs **40**). Compound **42**, with hydroxy substitution at the meta-position, showed weak affinity to all tested receptors. Compound **43** contains three substituents on the phenyl ring and demonstrated potent affinity to MOR (*K*_*i*_ = 4.3 nM), while its binding affinity to 5-HT_2A_ receptors was significantly weaker. As shown in Table 2, the SAR of the substituents on the side-chain phenyl ring is quite interesting and sensitive to minor structural changes. These data show that the receptor binding profiles of this class of compounds can be modulated by optimization of the substituents on this phenyl ring. *para*-Fluoro substitution on this ring offered compound **5** with potent and balanced binding to MOR and 5-HT_2A_ receptors, and possibly improved metabolic stability, so this structural feature was kept in the subsequent rounds of lead optimizations.

After studying the substituents on the side-chain phenyl ring, we evaluated the type of linkers between the tetracyclic core and the phenyl ring, as shown in Table 3. Replacing the oxygen on the linker with a methylene group provided compound **44**, which showed little change in binding affinity to the evaluated receptors relative to compound **5**. Such a structural change will further increase the hydrophobicity of the molecule, which is undesirable due to potentially increased nonspecific protein binding. Compound **45** containing a thioether linker exhibited similar binding affinity to MOR (*K*_*i*_ = 12.9 nM) and more potent affinity to 5-HT_2A_ receptors (*K*_*i*_ = 2.5 nM) relative to compound **5** containing an ether linker. It is known that thioether is redox-labile. After oxidation to sulfone, the resulting compound **46** only showed weak binding to 5-HT_2A_ receptors, without any affinity to other receptors. When the heteroatom on the linker was changed to nitrogen from oxygen, the resulting compound **47** maintained the affinity to MOR (*K*_*i*_ = 10.8 nM), but its binding to 5-HT_2A_ receptors was significantly weakened as compared to compound **5**. N-Methylation of **47** led to compound **48**, which demonstrated improved affinity to SERT and 5-HT_2A_ receptors at the expense of decreased affinity to MOR. Among these compounds, the ether linker offered compound **5** with potent affinity to the targeted receptors and potentially better metabolic stability than other evaluated linkers. Compound **5** has a ligand–lipophilicity efficiency (LLE) of 4.31, very close to the 4.43 mean LLE for approved oral drugs.^30^

In addition to the heteroatoms on the linkers in this class of compounds, the SAR of compounds with different side-chain lengths (*n* = 0, 1 or 2) were also studied, as summarized in Table 4. Compounds **49** and **50**, with ethyl ether linker (*n* = 0) and butyl ether linker (*n* = 2), respectively, exhibited weak-to-moderate binding affinity to all tested receptors, and the activities for both compounds were similar regardless of the linker length difference. However, compound **5**, containing the propyl ether linker (*n* = 1), showed remarkable improvement in binding affinity to MOR and 5-HT_2A_ receptors relative to **49** and **50**. These data suggest that the propyl ether linker provided optimal length and configuration to properly position both the key tetracyclic core and side-chain phenyl ring in the binding pockets of both receptors, and this linker has been kept in the subsequent lead optimizations.

We switched our focus to the exploration of the tetracyclic core after conducting the SAR study of the side chain. Various substituents, ring sizes, and stereochemistry were evaluated, as summarized in Tables 4–6. When a methyl group was substituted on the D-ring, the resulting compound **51** largely maintained binding affinity to 5-HT_2A_ receptors with a moderate increase in dopamine D_2_ receptor binding affinity, while its affinity for MOR and D_1_ receptors was decreased. Interestingly, the gem-dimethyl substituted analogue **52** showed further increased affinity to dopamine D_2_ receptors (*K*_*i*_ = 53 nM) relative to compound **5**, with affinity to 5-HT_2A_ receptors maintained (*K*_*i*_ = 8.6 nM) and activity at MOR and SERT largely diminished. Compound **52** could be a useful tool compound, considering its unique receptor binding profile.

As shown in Table 5, compounds **14** (*m* = 0) and **53** (*m* = 2) were synthesized, in addition to **5** (*m* = 1), to evaluate the impact of the size of the D-ring on receptor binding profiles. It is apparent that 5-HT_2A_ receptor affinity is insensitive to variations in the ring size, while MOR has little tolerance for ring size changes, and a six-membered D-ring is required for high-affinity binding to MOR. As for dopamine receptors, a five-membered D-ring appears to be preferred for D_2_ receptor binding affinity, while D_1_ receptors seem to show a slight preference to compound **5** with a six-membered D-ring. For SERT, compound **53**, with a seven-membered D-ring, showed slightly stronger inhibition than its analogues with smaller D-rings. Among these analogues, compound **5** exhibited the most potent binding affinity to MOR and 5-HT_2A_ receptors.

Before nominating compound **5** as our lead candidate, we further evaluated the stereochemistry of the two chiral carbons at the junction of the B and C rings. As shown in Table 6, stereoisomers **20** and **54** were evaluated, and the results were compared with **5**. It is quite interesting that **5** and its enantiomer **54** showed similar binding affinity to 5-HT_2A_ and dopamine D_1_ and D_2_ receptors, while **5** exhibited 26-fold more potent affinity to MOR than **54**. In addition, **54** did not show any inhibition of SERT, unlike **5**. Remarkably, the target binding profile of the racemic *trans* isomer **20** was quite different from **5**. It had the most potent binding affinity to dopamine D_1_ receptors (*K*_*i*_ = 5.6 nM), followed by MOR (*K*_*i*_ = 26 nM) and D_2_ receptors (*K*_*i*_ = 160 nM), yet it did not show any activity at 5-HT_2A_ receptors and SERT. Based upon the collected SAR, compound **5**, with absolute configuration of (6*bR*, 10*aS*) at the junction of the B and C rings, is certainly preferred.

In summary, through systematic optimization of the functional groups on the side chain, the heteroatom, and length of the linker, as well as the ring size, substituents, and stereochemistry of the tetracyclic core, lead compound **5** was identified. Further characterization of this development candidate was conducted in vitro and in vivo, as described in the next section.

### In Vitro and In Vivo Evaluation of ITI-333

The lead compound **5** (ITI-333) was further evaluated in additional receptor binding and functional assays. A broad screen of neurotransmitter receptors, enzymes, and channels revealed that compound **5** has potent binding affinity to serotonin 5-HT_2A_ (*K*_*i*_ = 8.3 nM), μ-opioid (*K*_*i*_ = 11 nM), adrenergic α_1A_ (*K*_*i*_ = 28 nM), and dopamine D_1_ (*K*_*i*_ = 50 nM) receptors, and moderate binding to dopamine D_2_ (*K*_*i*_ = 160 nM) receptors. There is no significant binding to any other studied targets, including δ, κ, or nociception opioid receptors. In cell-based functional assays, compound **5** was found to be a partial agonist at human recombinant MOR expressed in CHO-K1 cells, inhibiting cAMP accumulation elicited by forskolin with a maximal effect, or intrinsic efficacy, equal to 22% of that of the full MOR agonist DAMGO and an EC_50_ of 64 nM. Notably, compound **5** has no significant activity as a β-arrestin agonist (EC_50_ > 10 μM), yet it exhibited antagonistic activity in MOR-dependent β-arrestin signaling with an IC_50_ of 0.18 μM. Compound **5** was found to be an antagonist at human recombinant 5-HT_2A_ receptors, suppressing calcium (Ca^2+^) flux elicited by the 5-HT_2A_ agonist α-methylserotonin with an apparent dissociation constant (*K*_B_) of 9.5 nM. It was also found to be a very weak dopamine D_1_ receptor antagonist (*K*_B_ = 1.4 μM), blocking forskolin-induced cAMP accumulation in cells expressing human D_1_ receptors, but such weak functional activity at D_1_ receptors may not contribute to its pharmacology in vivo. Interestingly, compound **5** exhibited potent antagonist activity at adrenergic α_1A_ receptors (*K*_B_ = 7.7 nM), by inhibiting phospholipase C (PLC)-associated Ca^2+^ signaling. Various studies have shown that adrenergic α_1A_ antagonists are effective in the treatment of heroin, cocaine, and alcohol use disorders.^31−33^

Compound **5** is being developed as a tosylate salt, which is a crystalline, nonhygroscopic material with excellent stability and good physicochemical and biophysical properties, as summarized in Table 7. Human plasma protein binding was measured using a standard equilibrium dialysis technique, and compound **5** elicited 91.6% binding to human plasma protein. The pharmacokinetic (PK) properties of compound **5** have been extensively studied. A representative set of PK data are shown in Table 8. Compound **5** had a half-life of 9.6 h in monkeys after oral administration and exhibited good oral bioavailability (43.7%).

**Table 7:** Properties of the Tosylate Salt of Compound 5 (ITI-333)

Log *P* (neutral)	3.60
Log *P* (cationic)	0.66
p*K*_a_	7.75
kinetic solubility^a^	55.5 μg/mL
intrinsic solubility^a^	6.58 μg/mL
human plasma protein binding	91.6%

a Measured in 0.15 M KCl solution.

**Table 8:** Pharmacokinetic Properties of Compound **5** (ITI-333) after Intravenous and Oral Administration to Cynomolgus Monkeys

PK parameters^a^	PO^b^	IV^c^
*T*_max_ (h)	3	
*C*_max_ (ng/mL)	114	
*t*_1/2_ (h)	9.6	4.6
AUC_0-¥_ (area) (ng h/mL)	1408	1150
*V*_d_ (L/kg)		6.2
Cl (L/h/kg)		0.9
bioavailability (*F* %)	43.7%	

a *V*_d_, volume of distribution. Cl, clearance.

b Compound **5** was formulated in PEG-400 and dosed orally at 2.8 mg/kg.

c A solution of **5** in 45% Trappsol in deionized water with 1% DMSO was dosed intravenously at 1 mg/kg.

**Figure 2:**
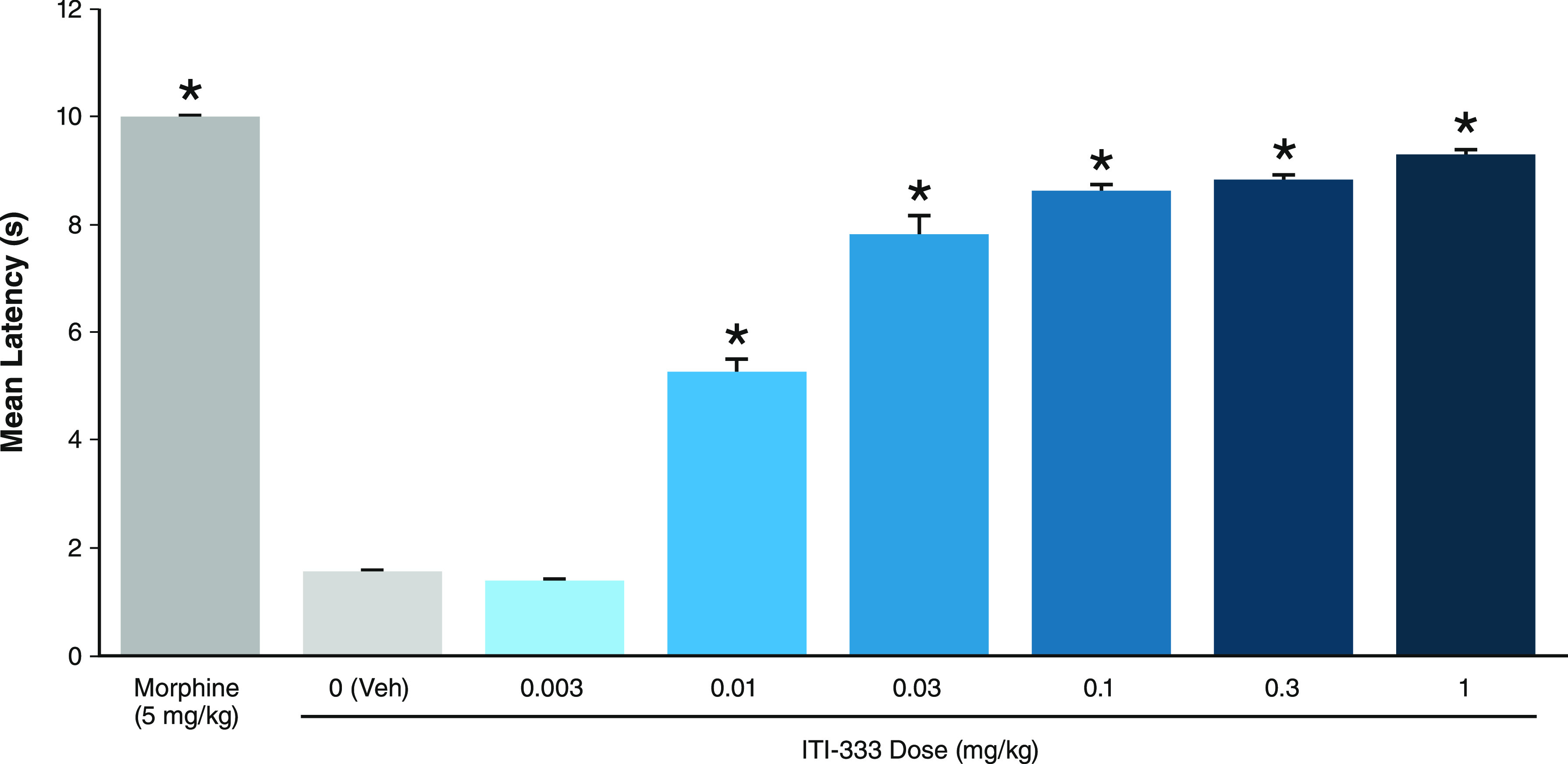
Compound **5** (ITI-333) dose-dependently increased latency to pain response. Mice (*n* = 10/group) were injected subcutaneously with either vehicle, ITI-333, or morphine 30 min prior to tail flick assay, which measured pain reflex threshold as the time (s) until mice responded with a tail flick movement away from a heat source. Data represent mean and SEM. *p < 0.05 vs vehicle.

The efficacy of compound **5** for the treatment of acute pain was evaluated in animal models. To evaluate the acute response to a nociceptive stimulus, the dose dependence of ITI-333 for analgesic efficacy in a tail flick (TF) assay was conducted in male CD-1 mice. The TF test measures the pain reflex threshold in restrained animals, a measure of acute nociception organized at the level of the spinal cord. Compound **5** (ITI-333) when administered alone, was found to dose-dependently increase latency to respond to a nociceptive stimulus (radiant heat) in this assay, indicative of an analgesic response, as shown in Figure 2. In this study, mice (*n* = 10/group) were treated with either vehicle, morphine, or ITI-333 30 min prior to tail flick assay. Acute subcutaneous administration of ITI-333 dose-dependently increased the latency to tail flick pain response compared to the vehicle with an ED_50_ of 0.016 mg/kg. The positive control, morphine (5 mg/kg), also significantly increased the latency to tail flick compared to the vehicle, as anticipated for a well-known analgesic.

**Figure 3:**
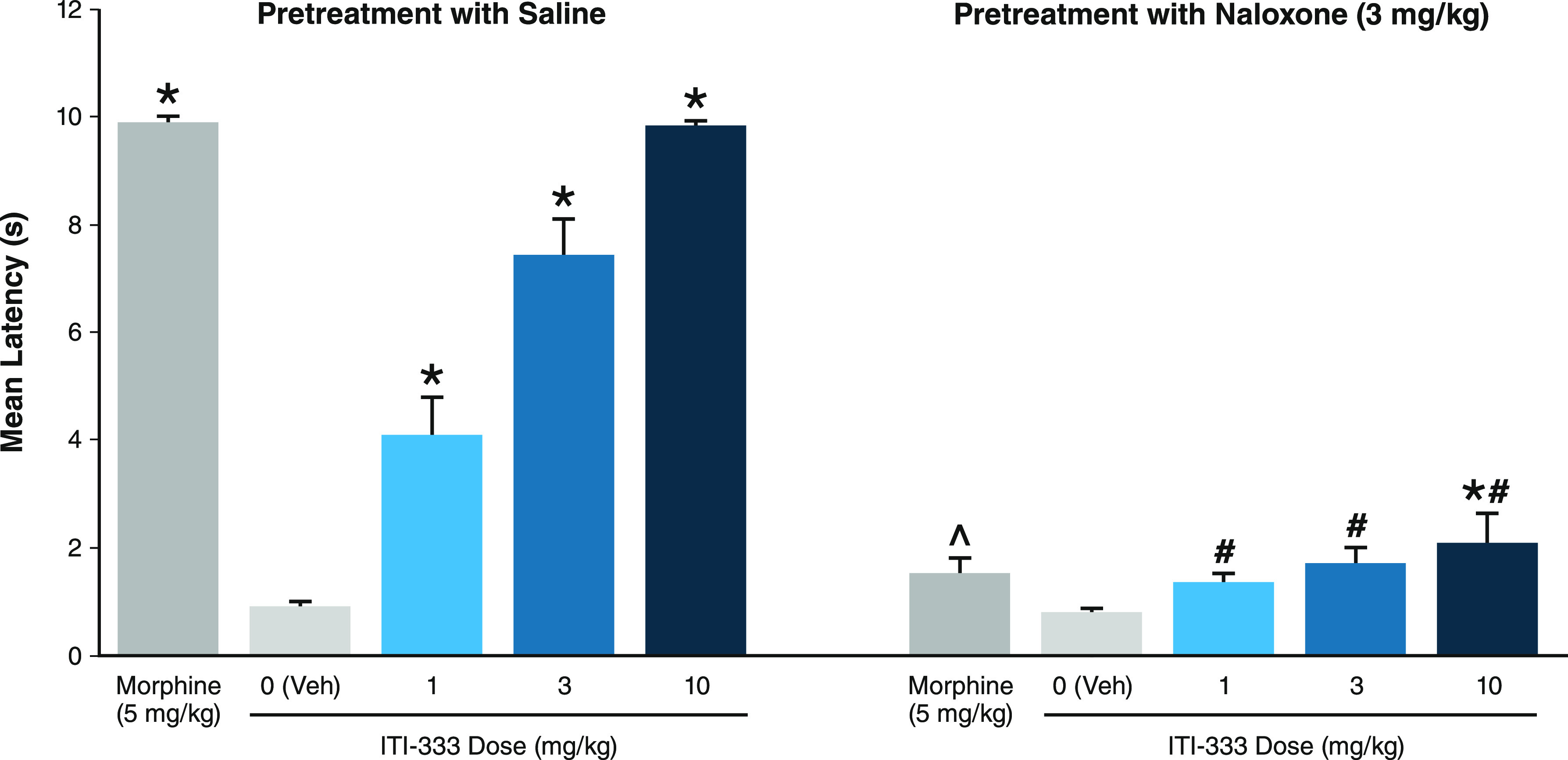
Analgesic effect of compound **5** (ITI-333) was attenuated by pretreatment with opioid antagonist naloxone. Mice (*n* = 9–10/group) were administered (IP) saline or naloxone and then, after 20 min, administered morphine (SC) or ITI-333 (PO) 30 min prior to tail flick assay, which measured pain reflex threshold as the time (s) until mice responded with a tail flick movement away from a heat source. Data represent mean and SEM. *p < 0.05 vs vehicle; ^∧^*p* < 0.05 vs morphine alone; ^#^*p* < 0.05 vs respective doses of ITI-333 alone. IP, intraperitoneal injection; PO, oral administration; SC, subcutaneous injection; Veh, vehicle.

To determine the dependence of ITI-333-induced analgesia on its μ-opioid receptor activity, compound **5** (ITI-333) was dosed orally in the presence and absence of the opioid antagonist, naloxone. As shown in Figure 3, when orally administered alone, ITI-333 dose-dependently increased latency to respond to a nociceptive stimulus in the TF assay. Yet, when the opioid antagonist naloxone (3 mg/kg) was administered to mice 20 min prior to ITI-333 or to the positive control morphine, the analgesic effects of both ITI-333 and morphine were attenuated. These data suggest the analgesic effects of ITI-333 require direct or indirect activation of μ-opioid receptors.

**Figure 4:**
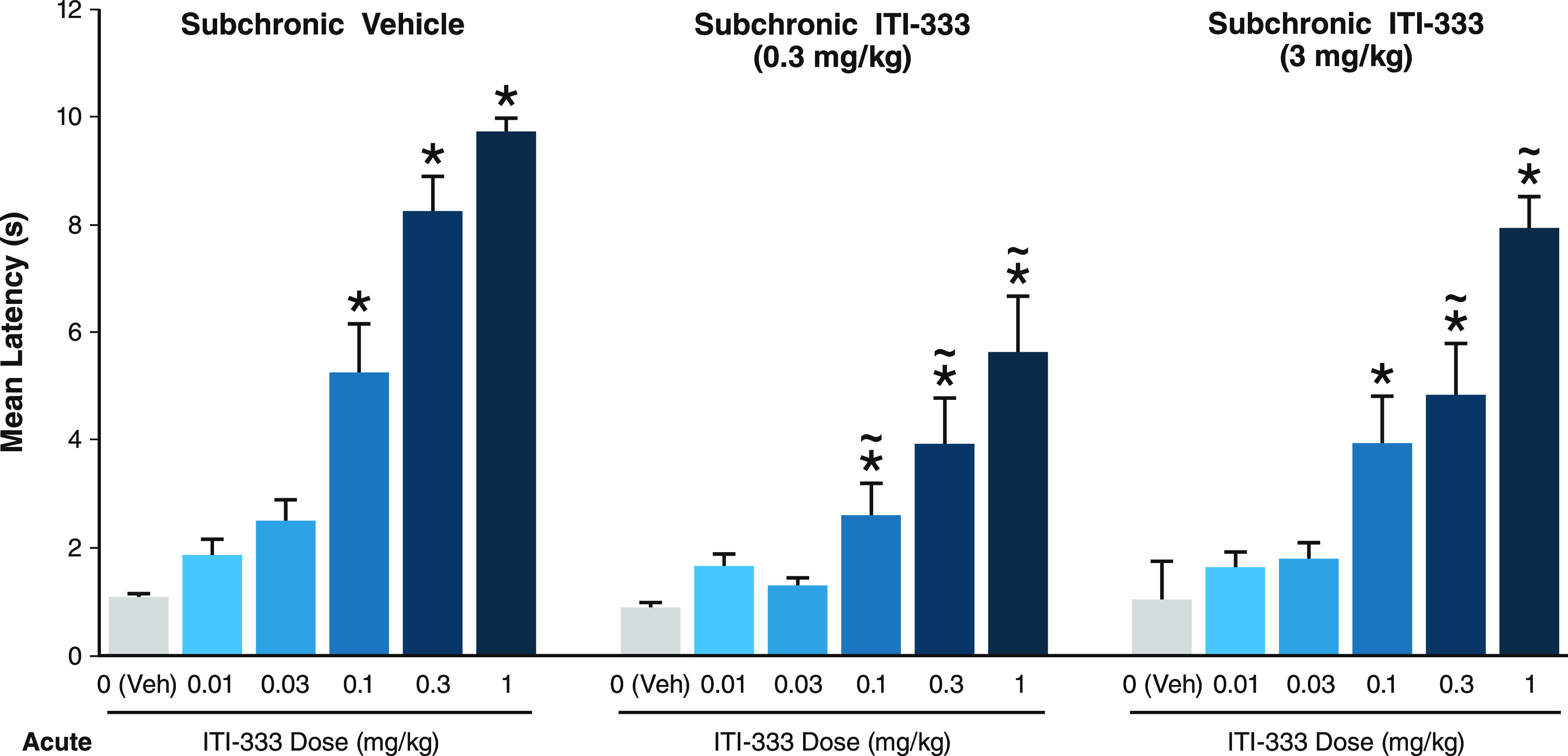
Effects of subchronic administration of compound **5** (ITI-333) on the acute analgesic effects of ITI-333. Mice (*n* = 8–10/group) were injected (SC) with vehicle or ITI-333 once daily for 14 days and then, on day 15, treated with ITI-333 or vehicle 30 min prior to tail flick assay, which measured pain reflex threshold as the time (s) until mice responded with a tail flick movement away from a heat source. Data represent mean and SEM. *p < 0.05 vs acute vehicle within each respective chronic treatment group; ∼*p* < 0.05 vs respective ITI-333 dose in chronic vehicle group. SC, subcutaneous injection; Veh, vehicle.

To determine the durability of the analgesic response to ITI-333, the effects of subchronic administration of ITI-333 on tail flick response were also studied. In the control group, mice were treated with the vehicle once daily for 14 days. Following subchronic vehicle treatment, the acute subcutaneous administration of ITI-333 (0.1, 0.3, and 1 mg/kg) again significantly increased the latency to response compared to the vehicle, as shown in Figure 4. Similarly, in mice subchronically treated with ITI-333 (0.3 or 3 mg/kg) once daily for 14 days, the acute administration of ITI-333 (0.1, 0.3, and 1 mg/kg) on day 15 also increased latency to tail flick compared to the respective vehicle. The acute effects of ITI-333 were maintained and were only slightly attenuated in mice subchronically treated with ITI-333 (0.3 or 3 mg/kg) compared to those treated with the vehicle.

**Figure 5:**
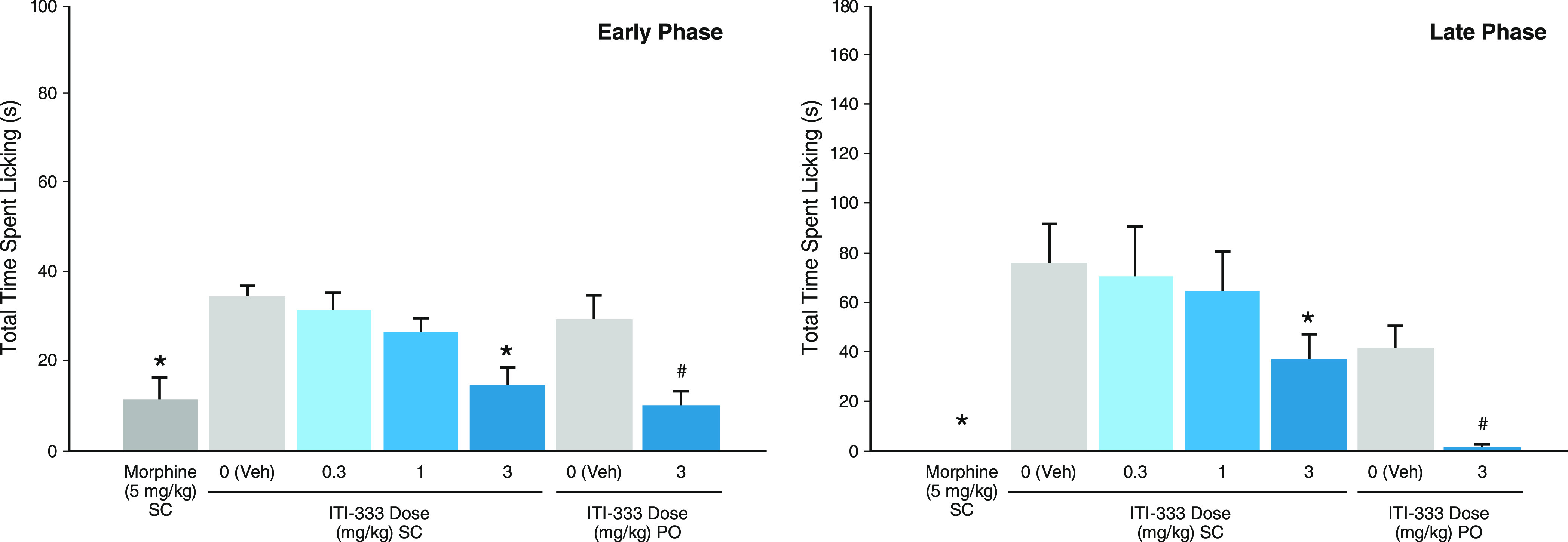
Analgesic effect of compound **5** (ITI-333) in mouse formalin paw assay for inflammatory pain. Mice (*n* = 10/group) were administered either vehicle, morphine, or ITI-333 (SC or PO, as indicated in figure legend) 30 min prior to the formalin paw assay, which measured the time each animal spent licking the inflamed paw during the acute (0–6 min, left) and late (7–40 min, right) phases. The minimum effective dose of ITI-333 in this model was 3 mg/kg for both SC and PO administration. Data represent mean ± SEM. *p < 0.05 compared to vehicle SC; ^#^*p* < 0.05 compared to vehicle PO. PO, oral administration; SC, subcutaneous injection.

The analgesic effects of compound **5** (ITI-333) were further evaluated in an inflammatory pain model. Pretreatment of mice with compound **5** (SC administration) dose-dependently reduced the licking response of mice to an inflammatory pain stimulus (i.e., formalin injection into the left hind paw). As shown in Figure 5, the positive control, morphine (5 mg/kg, SC), significantly suppressed licking behavior. Compound **5** showed significant suppression of licking behavior at a minimal effective dose (MED) of 3 mg/kg for both SC and oral (PO) administration during both the early/acute and the late/persistent phase of the inflammatory pain response.

## Conclusions

In this study, a novel class of tetracyclic pyridopyrroloquinoxalinone derivatives exhibiting potent binding affinity to serotonin 5-HT_2A_, μ-opioid receptors, and α_1A_ receptors was discovered. After extensive rounds of SAR studies and lead optimization, we identified compound **5** (ITI-333), a potent 5-HT_2A_ receptor antagonist and a partial agonist at MOR. Using a model (tail flick assay) to assess sensitivity to a nociceptive stimulus in mice, ITI-333 dose-dependently increased latency to respond to radiant heat following either oral or subcutaneous dosing indicative of analgesia. Similarly, ITI-333 also showed dose-dependent analgesic effects in an inflammatory pain model. Importantly, the analgesic activity of ITI-333 was blocked by the opioid antagonist, naloxone, and was retained following subchronic dosing, demonstrating the μ-opioid dependence and the durability of ITI-333-induced analgesia, respectively. Currently, this investigational new drug is in clinical development for the treatment of pain and other potential therapeutic indications.

## Experimental Section

### Chemistry

Unless otherwise noted, all materials were obtained from commercial suppliers and used without further purification. All reactions involving air- or moisture-sensitive reagents were performed under an argon atmosphere. All microwave-assisted reactions were conducted with an Initiator Eight EXP microwave system from Biotage (Charlotte, NC, USA). Silica gel or alumina column chromatography was performed with a CombiFlash Companion purification system from Teledyne ISCO (Lincoln, NE, USA). Filtrations were generally performed with Waterman paper filters. All products, unless otherwise noted, were purified by column chromatography and/or semipreparative reverse-phase HPLC. A Waters semipreparative HPLC system equipped with a Delta 600EF pump, 2996 PDA detector, and WFCIII fraction collector was used for compound purification using the following general HPLC method. Column: Gemini, AXIA packed, 10 μm C_18_ 110 Å, 250 mm × 21.2 mm; mobile phase A, 0.1% formic acid in water; mobile phase B, acetonitrile; gradient was adjusted and optimized based on compound polarity; HPLC run time was 22 min; flow rate was 24 mL/min; detection was at 210–350 nm. ^1^H NMR spectra were determined in the cited solvent on a Bruker DRX 300 or Avance III (400 or 500 MHz) spectrometer. ^13^C NMR spectra were recorded at 126 MHz. Chemical shifts are reported in delta (δ) units, parts per million (ppm) downfield from tetramethylsilane. Coupling constants are reported in hertz (Hz). Splitting patterns are designated as follows: s, singlet; br, broad singlet; d, doublet; t, triplet; q, quartet; m, multiplet. Purity of all final products was >95% as determined by reverse-phase UPLC/HPLC using method A and/or method B. Method A (UPLC): Waters ACQUITY UPLC system, ACQUITY HSS T3 column, 50 mm × 2.1 mm, 1.8 μm, 25 °C; mobile phase A, 0.1% formic acid in water/acetonitrile (95:5); mobile phase B, 0.1% formic acid in acetonitrile; gradient, 0.0–3.0 min, 5–95% B; 3.0–4.0 min, 95% B; 4.0–5.0 min, 95–5% B; flow rate 0.3 mL/min; detection at 210–400 nm. Method B (HPLC), Waters Alliance HT 2795 system, XSelect CSH C18 column, 100 × 4.6 mm, 2.5 μm, 15 °C; mobile phase A, 0.1% trifluoroacetic acid in water; mobile phase B, 0.1% trifluoroacetic acid in acetonitrile; gradient, 0.0–22.0 min, 5–27% B; 22–28 min, 27% B; 28–50 min, 27–70% B; flow rate 0.5 mL/min; detection at 230 nm. Mass spectral (MS) data were determined on a Micromass Quattro micro-API or LCT Premier XE mass spectrometer from Waters. High-resolution mass spectral (HRMS) data were determined on the LCT Premier XE mass spectrometer or Waters Xevo G2-XS QTOF. The chemical yields reported below are not optimized and correspond to specific examples of one preparation.

### Representative Synthetic Procedures of Polycyclic Pyrazolopyrimidine Derivatives Shown in Scheme 1

#### (6*bR*,10*aS*)-8-(3-(4-Fluorophenoxy)propyl)-6*b*,7,8,9,10,10*a*-hexahydro-1*H*-pyrido[3′,4′:4,5]pyrrolo[1,2,3-*de*]quinoxalin-2(3*H*)-one (**5**, ITI-333)

##### Step a: Ethyl (4*aS*,9*bR*)-5-(2-Amino-2-oxoethyl)-6-bromo-1,3,4,4*a*,5,9*b*-hexahydro-2*H*-pyrido[4,3-*b*]indole-2-carboxylate (**7**)

A mixture of ethyl (4*aS*,9*bR*)-6-bromo-1,3,4,4*a*,5,9*b*-hexahydro-2*H*-pyrido[4,3-*b*]indole-2-carboxylate (compound **6**, 21.5 g, 66.2 mmol), chloroacetamide (9.3 g, 100 mmol), and KI (17.7 g, 107 mmol) in anhydrous dioxane (60 mL) was heated under an argon atmosphere at 104 °C for 48 h. The reaction was cooled to room temperature, and the solvent was removed under reduced pressure. The residue was suspended in DCM (200 mL) and washed with water (100 mL). The DCM phase was separated, dried over K_2_CO_3_ and concentrated to give a brown oil. This oil product was suspended in ethyl acetate (100 mL), sonicated for 5 min, and allowed to stand at room temperature for 2 h. The precipitate was filtered, and the filtered cake was rinsed with ethyl acetate (2 mL) and dried under high vacuum. The title compound **7** was given as an off-white solid (19 g, 75% yield), which was directly used in the next reaction without further purification. ^1^H NMR (500 MHz, DMSO-*d*_6_): δ 7.36 (s, 1H), 7.16 (dd, *J* = 8.0, 1.2 Hz, 1H), 7.09–6.96 (m, 2H), 6.57 (dd, *J* = 8.0, 7.2 Hz, 1H), 4.22 (d, *J* = 18.1 Hz, 1H), 4.07–3.88 (m, 3H), 3.83 (m, 1H), 3.66 (s, 1H), 3.59–3.45 (m, 1H), 1.90 (m, 1H), 1.80 (s, 1H), and 1.15 (t, *J* = 7.0 Hz, 3H). HRMS (ESI) *m*/*z* calcd for C_16_H_21_BrN_3_O_3_ [M + H]^+^, 382.0761; found: 382.0771.

##### Step b: Ethyl (6*bR*,10*aS*)-2-Oxo-2,3,6*b*,9,10,10*a*-hexahydro-1*H*-pyrido[3′,4′:4,5]pyrrolo[1,2,3-*de*]quinoxaline-8(7*H*)-carboxylate (**8**)

A suspension of ethyl (4*aS*,9*bR*)-6-bromo-1,3,4,4*a*,5,9*b*-hexahydro-2*H*-pyrido[4,3-*b*]indole-2-carboxylate (**7**) (12.9 g, 33.7 mmol), KI (10.6 g, 63.8 mmol), and CuI (1.34 g, 6.74 mmol) dioxane (50 mL) was bubbled with argon for 5 min, and *N*,*N*,*N*′,*N*′-tetramethylethylenediamine (3 mL) was added. After stirring at 100 °C for 48 h, the reaction mixture was cooled to room temperature and then poured onto a silica gel pad to filter. The filtered cake was rinsed with ethyl acetate (2× 250 mL), and the filtrate was concentrated to give the title compound **8** as a white solid (8 g, 79% yield). This crude product was directly used in the next reaction without further purification. ^1^H NMR (500 MHz, DMSO-*d*_6_): δ 10.39 (s, 1H), 6.82 (d, *J* = 7.2 Hz, 1H), 6.66 (t, *J* = 7.5 Hz, 1H), 6.60 (dd, *J* = 7.8, 1.0 Hz, 1H), 4.04 (m, 2H), 3.92 (s, 2H), 3.85 (d, *J* = 14.6 Hz, 1H), 3.73–3.61 (m, 1H), 3.47 (m, 1H), 3.39 (d, *J* = 14.5 Hz, 1H), 3.32–3.23 (m, 1H), 3.12 (s, 1H), 2.76 (d, *J* = 46.5 Hz, 1H), 1.95 (dd, *J* = 14.6, 3.4 Hz, 1H), 1.82 (s, 0H), and 1.18 (t, *J* = 7.1 Hz, 3H). HRMS (ESI) *m*/*z* calcd for C_16_H_20_N_3_O_3_ [M + H]^+^, 302.1499; found: 302.1499.

##### Step c: (6*bR*,10*aS*)-6*b*,7,8,9,10,10*a*-Hexahydro-1*H*-pyrido[3′,4′:4,5]pyrrolo[1,2,3-*de*]quinoxalin-2(3*H*)-one (**9**)

Ethyl (6*bR*,10*aS*)-2-oxo-2,3,6*b*,9,10,10*a*-hexahydro-1*H*-pyrido[3′,4′:4,5]pyrrolo[1,2,3-*de*]quinoxaline-8(7*H*)-carboxylate (6.4 g, 27.9 mmol) was suspended in HBr acetic acid solution (64 mL, 33% w/w) at room temperature and heated to 50 °C under stirring. After stirring at 50 °C for 8 h, the reaction was cooled to room temperature and ethyl acetate (300 mL) was added. The precipitate was filtered, and the filtered cake was washed with ethyl acetate (10 mL) and dried under vacuum. The obtained HBr salt was suspended in methanol (100 mL) and cooled to temperature < 5 °C using isopropanol/dry ice. To this cooled suspension, under stirring, ammonia solution (20 mL, 7 N in methanol) was slowly added until the solution pH was 14. This solution was evaporated to dryness, and the residue was dried over high vacuum to give the title compound **9** (7.8 g, >99% yield). This crude product was used directly in the next step without any further purification. ^1^H NMR (500 MHz, DMSO-*d*_6_): δ 10.37 (s, 1H), 6.76 (d, *J* = 7.3 Hz, 1H), 6.64 (t, *J* = 7.5 Hz, 1H), 6.59 (d, *J* = 7.7 Hz, 1H), 3.82 (d, *J* = 14.5 Hz, 1H), 3.36–3.28 (m, 3H), 3.09 (m, 1H), 3.00 (m, 1H), 2.77 (m, 1H), 2.63 (t, *J* = 12.7 Hz, 1H), 2.21 (t, *J* = 11.5 Hz, 1H), 1.89 (d, *J* = 14.5 Hz, 1H), and 1.77–1.66 (m, 1H). HRMS (ESI) *m*/*z* calcd for C_13_H_16_N_3_O [M + H]^+^, 230.1288; found: 230.1279.

##### Step d: (6*bR*,10*aS*)-8-(3-(4-Fluorophenoxy)propyl)-6*b*,7,8,9,10,10*a*-hexahydro-1*H*-pyrido[3′,4′:4,5]pyrrolo[1,2,3-*de*]quinoxalin-2(3*H*)-one (**5**; R_1_ = 4-FPhOCH_2_CH_2_CH_2_)

A mixture of compound **9** (1.0 g, 4.4 mmol), 1-(3-chloroproxy)-4-fluoro-benzene (1.4 mL, 8.8 mmol), and KI (1.45 g, 8.7 mmol) in DMF (20 mL) was bubbled with argon for 3 min, and DIPEA (1.50 mL, 8.7 mmol) was added. The mixture was heated to 78 °C stirred for 2 h, and then cooled to room temperature. The solvent was removed, and the residue was dissolved in DCM (30 mL) and washed with water (20 mL). The DCM phase was dried over K_2_CO_3_, filtered, and the filtrate was concentrated. The obtained product was purified with silica gel column chromatography using a gradient of 0–55% mixture of ethyl acetate/methanol/7 N NH_3_ in methanol (10:1:0.1 v/v/v) in ethyl acetate as an eluent. The eluted product was dissolved in methanol (5 mL) and allowed to stand at room temperature for 30 min. The precipitate was filtered and dried under vacuum to give the final product **5** as a white solid (0.63 g, 38% yield) ^1^H NMR (500 MHz, DMSO-*d*_6_): δ 10.34 (s, 1H), 7.11–7.07 (m, 2H), 6.94–6.90 (m, 2H), 6.77–6.76 (m, 1H), 6.63 (t, *J* = 7.5 Hz, 1H), 6.58 (dd, *J* = 7.7, 1.1 Hz, 1H), 3.97 (t, *J* = 6.4 Hz, 2H), 3.80 (d, *J* = 14.6 Hz, 1H), 3.31 (s, 1H), 3.27–3.17 (m, 2H), 2.88–2.84 (m, 1H), 2.66–2.61 (m, 1H), 2.43–2.32 (m, 2H), 2.12–2.07 (m, 1H), 1.96–1.91 (m, 1H), 1.87–1.76 (m, 3H), and 1.68 (t, *J* = 11.0 Hz, 1H). ^13^C NMR (126 MHz, DMSO-*d*_6_): δ 166.1, 156.4 (d, *J* = 235.6 Hz), 155.0, 137.5, 130.5, 123.8, 120.0, 117.8, 115.7 (d, *J* = 27.7 Hz), 115.6 (d, *J* = 2.52 Hz), 112.2, 66.4, 65.6, 56.3, 54.4, 51.6, 48.7, 41.1, 26.3, and 24.3. HRMS (ESI) *m*/*z* calcd for C_22_H_25_FN_3_O_2_ [M + H]^+^, 382.1925; found: 382.1923.

### Synthetic Procedure of Compound **14** as Shown in Scheme 2

#### Step a: (4*aS*,9*bR*)-6-Bromo-2-(3-(4-fluorophenoxy)propyl)-2,3,4,4*a*,5,9*b*-hexahydro-1*H*-pyrido[4,3-*b*]indole (**11**)

A mixture of compound **10** (5.0 g, 19.7 mmol), 1-(3-chloroproxy)-4-fluoro-benzene (3.73 mL, 23.7 mmol), and KI (6.56 g, 39.5 mmol) in DMF (12 mL) was bubbled with argon for 3 min, and DIPEA (6.88 mL, 39.5 mmol) was added. The mixture was heated to 80 °C, stirred for 2 h, and then cooled to room temperature. The solvent was removed, and the residue was purified with silica gel column chromatography using a gradient of 0–55% mixture of ethyl acetate/methanol/7 N NH_3_ in methanol (10:1:0.1 v/v/v) in ethyl acetate as an eluent. The title compound **11** was obtained as a pale white solid (7.6 g, 95% yield). ^1^H NMR (500 MHz, DMSO-*d*_6_): δ 7.17–7.07 (m, 3H), 7.03 (d, *J* = 7.1 Hz, 1H), 6.98–6.87 (m, 2H), 6.51 (t, *J* = 8.1, 1H), 5.55 (d, *J* = 2.4 Hz, 1H), 3.96 (t, *J* = 6.3 Hz, 2H), 3.73 (m, 1H), 3.17 (m, 1H), 2.82 (d, *J* = 79.5 Hz, 1H), 2.65 (m, 1H), 2.45–2.33 (m, 3H), 2.32–2.22 (m, 1H), 2.08 (dd, *J* = 11.5, 8.8 Hz, 1H), and 1.92–1.70 (m, 3H). HRMS (ESI) *m*/*z* calcd for C_20_H_23_BrFN_2_O [M + H]^+^, 405.0972; found: 405.0992.

#### Step b: (4*aS*,9*bR*)-2-(3-(4-Fluorophenoxy)propyl)-2,3,4,4*a*,5,9*b*-hexahydro-1*H*-pyrido[4,3-*b*]indol-6-amine (**12**)

*N*,*N*-Dimethylacetamide (6.0 mL) was added to a mixture of copper iodide (0.376 g, 1.97 mmol), cesium carbonate (6.43 g, 19.7 mmol), and compound **11** (4.0 g, 9.87 mmol) under room temperature, then the reaction mixture was bubbled with argon for 5 min and sealed. 2,2,6,6-Tetramethyl-3,5-heptanedione (2.06 mL, 9.87 mmol) and ammonium hydroxide solution (7.0 mL, 59.2 mmol) were added via a syringe. Then the mixture was reacted under microwave irradiation at 95 °C, 20 W, for 4.5 h. After the mixture was cooled to room temperature, the solvent was evaporated, and the residue was directly purified by column chromatography on silica gel to afford the desired product (2.1 g, 62% yield) as a black solid. ^1^H NMR (500 MHz, DMSO-*d*_6_): δ 8.19 (d, *J* = 1.8 Hz, 2H), 7.15–7.04 (m, 2H), 6.98–6.88 (m, 2H), 6.40 (d, *J* = 5.6 Hz, 2H), 6.37–6.33 (m, 1H), 3.97 (m, 2H), 3.67 (m, 1H), 3.05–3.00 (m, 1H), 2.69 (m, 1H), 2.59–2.52 (m, 1H), 2.48–2.34 (m, 4H), 2.12 (t, *J* = 11.8, 1H), 1.87 (m, 3H), and 1.73 (m, 1H). HRMS (ESI) *m*/*z* calcd for C_20_H_25_FN_3_O [M + H]^+^, 342.1976; found: 342.1984.

#### Step c: Ethyl ((4*aS*,9*bR*)-2-(3-(4-Fluorophenoxy)propyl)-2,3,4,4*a*,5,9*b*-hexahydro-1*H*-pyrido[4,3-*b*]indol-6-yl)carbamate (**13**)

To a solution of (4*aS*,9*bR*)-2-(3-(4-fluorophenoxy)propyl)-2,3,4,4a,5,9*b*-hexahydro-1*H*-pyrido[4,3-*b*]indol-6-amine (0.30 g, 0.88 mmol) and DMAP (0.107 g, 0.88 mmol) in pyridine (3 mL) at 0 °C was added ethyl chloroformate (0.084 mL, 0.88 mmol), and the mixture was stirred for 1 h. The reaction was quenched with water (6 mL) and extracted with CH_2_Cl_2_ (15 mL). The separated CH_2_Cl_2_ phase was washed with water (10 mL) and evaporated to dryness. The obtained crude product (0.363 mg, yield: 99%) was used directly in the next reaction without further purification. HRMS (ESI) *m*/*z* calcd for C_23_H_29_FN_3_O_3_ [M + H]^+^, 414.2187; found: 414.2190.

#### Step d: (5*bR*,9*aS*)-7-(3-(4-Fluorophenoxy)propyl)-5b,6,7,8,9,9*a*-hexahydroimidazo[4,5,1-*hi*]pyrido[4,3-*b*]indol-1(2*H*)-one (**14**)

A 25 mL round-bottom flask was charged with ethyl ((4*aS*,9*bR*)-2-(3-(4-fluorophenoxy)propyl)-2,3,4,4*a*,5,9*b*-hexahydro-1*H*-pyrido[4,3-*b*]indol-6-yl)carbamate (0.360 mg, 0.87 mmol), Cs_2_CO_3_ (2.12 g, 6.50 mmol), and toluene (8 mL) under an argon atmosphere. Into this suspension was dropped 1 M lithium bis(trimethylsilyl)amide in THF (2.61 mL, 2.61 mmol), and the mixture was heated to 75 °C and stirred for 0.5 h. After cooling to room temperature, the reaction mixture was quenched with water (0.5 mL) and evaporated to dryness. The residue was purified on a silica gel column to provide the title compound as an off-white solid (0.163 g, 51% yield). ^1^H NMR (500 MHz, DMSO-*d*_6_): δ 10.33 (s, 1H), 7.34–7.02 (m, 2H), 6.93–6.86 (m, 2H), 6.84–6.79 (m, 2H), 6.71 (dd, *J* = 5.6, 2.5 Hz, 1H), 4.57 (q, *J* = 5.8 Hz, 1H), 3.92–3.87 (m, 3H), 2.88 (dd, *J* = 12.1, 5.7 Hz, 1H), 2.57 (dd, *J* = 12.1, 7.1 Hz, 1H), 2.47–2.42 (m, 3H), 2.40–2.35 (m, 1H), 2.14–2.07 (m, 1H), and 1.90–1.81 (m, 3H). ^13^C NMR (126 MHz, MeOD-*d*_4_): δ 158.9 (d, *J* = 204.1 Hz), 157.8, 156.3, 141.9, 125.6, 124.9, 123.9, 117.6, 116.8, 116.7 (d, *J* = 12.6 Hz), 110.1, 67.0, 61.6, 56.0, 54.1, 47.6, 26.3, and 25.7. HRMS (ESI) *m*/*z* calcd for C_21_H_23_FN_3_O_2_ [M + H]^+^, 368.1769; found: 368.1768.

### Synthetic Procedure of Racemic Compound **20** as Shown in Scheme 3

#### Step a: (±)-(*trans*)-2-Benzyl-6-bromo-2,3,4,4*a*,5,9*b*-hexahydro-1*H*-pyrido[4,3-*b*]indole ((±)-*trans*-**16**)

To a suspension of 2-benzyl-6-bromo-2,3,4,5-tetrahydro-1*H*-pyrido[4,3-*b*]indole (5.50 g, 16 mmol) in THF (10 mL) was slowly added borane (1.0 M in THF, 150 mL) at room temperature. The resulting solution was heated to 40 °C and stirred for 72 h. After cooling to room temperature, the reaction mixture was concentrated, and HCl (6 N, 37 mL) was added. The mixture was heated to 100 °C, stirred for 0.5 h, and then cooled to room temperature. Water (45 mL) was added, and the precipitate was filtered. The filtrate was basified and concentrated. The residue was purified with column chromatography, using a gradient of 0–100% mixed solvents [ethyl acetate/methanol/7 N NH_3_ (10:1:0.1 v/v/v)] in ethyl acetate. The title compound was obtained as a white foam (1.56 g, 28% yield). ^1^H NMR (500 MHz, DMSO-*d*_6_): δ 7.34 (d, *J* = 5.8 Hz, 4H), 7.28 (dt, *J* = 5.9, 2.2 Hz, 1H), 7.16 (dd, *J* = 8.2, 1.1 Hz, 1H), 6.96 (d, *J* = 7.2 Hz, 1H), 6.56 (dd, *J* = 8.1, 7.2 Hz, 1H), 5.77 (d, *J* = 3.0 Hz, 1H), 3.65 (s, 2H), 3.49–3.37 (m, 2H), 3.01–2.89 (m, 1H), 2.85–2.69 (m, 1H), 2.20 (m, 1H), 2.16–2.05 (m, 1H), 2.05–1.98 (m, 1H), and 1.86–1.66 (m, 1H). HRMS (ESI) *m*/*z* calcd for C_18_H_20_BrN_2_ [M + H]^+^, 343.0804; found: 343.0805.

#### Step b: (±)-(*trans*)-2-(2-Benzyl-6-bromo-1,2,3,4,4*a*,9*b*-hexahydro-5*H*-pyrido[4,3-*b*]indol-5-yl)acetamide ((±)-*trans*-**17**)

To a degassed suspension of (±)-(*trans*)-2-benzyl-6-bromo-2,3,4,4*a*,5,9*b*-hexahydro-1*H*-pyrido[4,3-*b*]indole (0.420 g, 1.22 mmol) in toluene (4 mL) at 0 °C was added lithium bis(trimethylsilyl)amide (1.0 M in toluene, 4 mL), and the mixture was stirred for 0.5 h. Chloroacetamide (0.112 g, 1.22 mmol) was added, and the mixture was stirred at room temperature overnight. The reaction mixture was quenched with methanol (5 mL) and concentrated. The residue was suspended in dichloromethane (100 mL), washed with water (20 mL), and dried over MgSO_4_. The dichloromethane phase was separated and evaporated to dryness. The residue was purified with column chromatography using a gradient of 0–50% mixed solvents [ethyl acetate/methanol/7 N NH_3_ (10:1:0.1 v/v/v)] in ethyl acetate. The title compound was obtained as a white solid (0.340 g, yield 69%). ^1^H NMR (500 MHz, DMSO-*d*_6_): δ 7.49 (s, 2H), 7.32 (m, 4H), 7.29–7.22 (m, 1H), 6.99–6.93 (m, 2H), 6.58 (m, 1H), 4.18–3.86 (m, 2H), 3.73–3.52 (m, 2H), 3.46 (m, 1H), 3.42 (m, 1H), 3.04–2.92 (m, 1H), 2.75 (m, 1H), 2.15 (t, *J* = 10.5 Hz, 1H), 2.11–2.00 (m, 1H), 1.95 (m, 1H), and 1.66 (dd, *J* = 11.7, 4.0 Hz, 1H). HRMS (ESI) *m*/*z* calcd for C_20_H_23_BrN_3_O [M + H]^+^, 400.1019; found: 400.1010.

#### Step c: (±)-(*trans*)-8-Benzyl-6*b*,7,8,9,10,10*a*-hexahydro-1*H*-pyrido[3′,4′:4,5]pyrrolo[1,2,3-*de*]quinoxalin-2(3*H*)-one ((±)-*trans*-**18**)

A mixture of (±)-(*trans*)2-(2-benzyl-6-bromo-1,2,3,4,4*a*,9*b*-hexahydro-5*H*-pyrido[4,3-*b*]indol-5-yl)acetamide (0.92 g, 2.3 mmol), K_2_CO_3_ (0.69 g, 5.1 mmol), and CuI (0.11 g, 0.58 mmol) in dioxane (10 mL) was bubbled with argon for 5 min. To this mixture was added *N*,*N*,*N*′,*N*′-tetramethylethylenediamine (0.20 mL), and the resulting suspension was stirred at 100 °C for 48 h. The mixture was cooled to room temperature and poured onto a silica gel pad to filter. The filtered cake was rinsed with ethyl acetate/methanol/7 N NH_3_ (20:1:0.1 v/v/v) (200 mL). The filtrate was concentrated to dryness to give the title compound as a white solid (0.157 g, 21% yield). This crude product was directly used in the next reaction without further purification. ^1^H NMR (500 MHz, DMSO-*d*_6_): δ 10.35 (s, 1H), 7.35 (d, *J* = 5.5 Hz, 4H), 7.27 (m, 1H), 6.86–6.64 (m, 2H), 6.60 (dt, *J* = 7.6, 1.1 Hz, 1H), 3.78 (d, *J* = 14.4 Hz, 1H), 3.70–3.54 (m, 2H), 3.51–3.41 (m, 1H), 2.98 (dt, *J* = 11.8, 3.3 Hz, 1H), 2.84 (m, 1H), 2.51 (m, 2H), 2.30–2.09 (m, 2H), 2.04 (dd, *J* = 11.8, 3.1 Hz, 1H), and 1.80–1.61 (m, 1H). HRMS (ESI) *m*/*z* calcd for C_20_H_22_N_3_O [M + H]^+^, 320.1757; found: 320.1745.

#### Step d: (±)-(*trans*)-6*b*,7,8,9,10,10*a*-Hexahydro-1*H*-pyrido[3′,4′:4,5]pyrrolo[1,2,3-*de*]quinoxalin-2(3*H*)-one ((±)-*trans*-**19**)

Pd/C (0.011 g) was added to a solution (45 mL) of (±)-(*trans*)-8-benzyl-6*b*,7,8,9,10,10*a*-hexahydro-1*H*-pyrido[3′,4′:4,5]pyrrolo[1,2,3-*de*]-quinoxalin-2(3*H*)-one (0.050 g, 0.16 mmol) in methanol (45 mL). The mixture was degassed with hydrogen and stirred under a hydrogen atmosphere overnight. Methanol was removed, and the residue was purified with column chromatography, using a gradient of 0–100% mixed solvents [ethyl acetate/methanol/7 N NH_3_ (10:1:0.1 v/v/v)]. The title compound was given as a white solid (0.021 g, 57% yield). HRMS (ESI) *m*/*z* calcd for C_13_H_16_N_3_O [M + H]^+^, 230.1288; found: 320.1286.

#### Step e: (±)-(*trans*)-8-(3-(4-Fluorophenoxy)propyl)-6*b*,7,8,9,10,10*a*-hexahydro-1*H*-pyrido[3′,4′:4,5]pyrrolo[1,2,3-*de*]quinoxalin-2(3*H*)-one ((±)-*trans*-**20**)

A mixture of (±)-(*trans*)-6*b*,7,8,9,10,10*a*-hexahydro-1*H*-pyrido[3′,4′:4,5]pyrrolo[1,2,3-*de*]quinoxalin-2(3*H*)-one (0.021 g, 0.09 mmol), 1-(3-chloropropoxy)-4-fluorobenzene (25 μL, 0.14 mmol), and KI (0.023 g, 0.14 mmol) in DMF (3.0 mL) was bubbled with argon for 3 min, and DIPEA (25 μL, 0.14 mmol) was added. The resulting mixture was heated to 76 °C and stirred at this temperature for 2 h. The solvent was removed, and the residue was purified by silica gel column chromatography using a gradient of 0–100% mixed solvents [ethyl acetate/methanol/7 N NH_3_ (10:1:0.1 v/v/v)] in ethyl acetate. The title product was obtained as a white solid (0.0035 g, 10% yield). ^1^H NMR (500 MHz, DMSO-*d*_6_): δ 10.37 (s, 1H), 7.11 (t, *J* = 8.8 Hz, 2H), 7.00–6.91 (m, 2H), 6.79 (d, *J* = 7.4 Hz, 1H), 6.70 (t, *J* = 7.6 Hz, 1H), 6.60 (d, *J* = 7.8 Hz, 1H), 4.00 (t, *J* = 6.4 Hz, 2H), 3.79 (d, *J* = 14.4 Hz, 1H), 3.53 (dd, *J* = 10.6, 3.4 Hz, 1H), 3.31 (d, *J* = 14.4 Hz, 2H), 3.05 (d, *J* = 11.3 Hz, 1H), 2.86–2.71 (m, 1H), 2.58 (t, *J* = 6.6 Hz, 2H), 2.18–2.00 (m, 3H), 1.92 (p, *J* = 6.7 Hz, 2H), and 1.69 (dd, *J* = 11.6, 4.0 Hz, 1H). ^13^C NMR (126 MHz, DMSO-*d*_6_): δ 166.1, 156.4 (d, *J* = 235.6 Hz), 155.0, 138.5, 129.1, 124.1, 120.5, 116.5, 115.7 (d, *J* = 26.5 Hz), 115.6 (d, *J* = 5.0 Hz), 112.3, 76.3, 66.4, 54.4, 53.9, 52.8, 51.7, 47.0, 28.5, and 26.6. HRMS (ESI) *m*/*z* calcd for C_22_H_25_FN_3_O_2_ [M + H]^+^, 382.1925; found: 382.1942.

Compounds **21**–**51, 53**–**54** were prepared in an analogous fashion following the procedure described in the synthesis of compound **5**.

#### (6*bR*,10*aS*)-8-Butyl-6*b*,7,8,9,10,10*a*-hexahydro-1*H*-pyrido[3′,4′:4,5]pyrrolo[1,2,3-*de*]quinoxalin-2(3*H*)-one (**21**)

The synthesis method was analogous to the synthesis of compound **5** in Scheme 1 wherein 1-chlorobutane was added in step d instead of 1-(3-chloropropoxy)-4-fluorobenzene. 37% isolated yield. ^1^H NMR (500 MHz, DMSO-*d*_6_): δ 10.34 (s, 1H), 6.77 (dd, *J* = 7.4, 1.0 Hz, 1H), 6.64 (t, *J* = 7.5 Hz, 1H), 6.58 (dd, *J* = 7.9, 1.0 Hz, 1H), 3.80 (d, *J* = 14.5 Hz, 1H), 3.30–3.23 (m, 2H), 3.22–3.17 (m, 1H), 2.85–2.81 (m, 1H), 2.63–2.58 (m, 1H), 2.30–2.13 (m, 2H), 2.11–2.00 (m, 1H), 1.94–1.91 (m, 1H), 1.82–1.74 (m, 1H), 1.63 (t, *J* = 11.0 Hz, 1H), 1.46–1.34 (m, 2H), 1.33–1.22 (m, 2H), and 0.87 (t, *J* = 7.3 Hz, 3H). ^13^C NMR (126 MHz, DMSO-*d*_6_): δ 166.1, 137.5, 130.5, 123.8, 119.9, 117.8, 112.2, 65.7, 57.6, 56.4, 51.6, 48.7, 41.1, 28.6, 24.3, 20.1, and 13.9. HRMS (ESI) *m*/*z* calcd for C_17_H_24_N_3_O [M + H]^+^, 286.1914; found: 286.1914.

#### (6*bR*,10*aS*)-8-(3-Hydroxypropyl)-6*b*,7,8,9,10,10*a*-hexahydro-1*H*-pyrido[3′,4′:4,5]pyrrolo[1,2,3-*de*]quinoxalin-2(3*H*)-one (**22**)

The synthesis method was analogous to the synthesis of compound **5** in Scheme 1 wherein 3-chloro-1-propanol was added in step d instead of 1-(3-chloropropoxy)-4-fluorobenzene. 2.8% isolated yield. ^1^H NMR (500 MHz, DMSO-*d*_6_): δ 10.38 (s, 1H), 6.79 (dd, *J* = 7.3, 1.0 Hz, 1H), 6.65 (t, *J* = 7.6 Hz, 1H), 6.59 (dd, *J* = 7.8, 1.0 Hz, 1H), 3.82 (d, *J* = 14.5 Hz, 1H), 3.44 (t, *J* = 6.3 Hz, 2H), 3.30–3.21 (m, 4H), 2.93–2.89 (m, 1H), 2.71–2.68 (m, 1H), 2.42–2.31 (m, 2H), 2.17–2.11 (m, 1H), 1.99–1.95 (m, 1H), 1.84–1.77 (m, 1H), 1.71 (t, *J* = 11.0 Hz, 1H), and 1.59 (p, *J* = 6.8 Hz, 2H). ^13^C NMR (126 MHz, MeOD-*d*_4_): δ 169.7, 138.9, 129.5, 125.4, 122.4, 119.9, 114.6, 66.1, 60.5, 56.3, 55.1, 52.7, 49.2, 41.0, 28.4, and 23.4. HRMS (ESI) *m*/*z* calcd for C_16_H_22_N_3_O_2_ [M + H]^+^, 288.1707; found: 288.1704.

#### (6*bR*,10*aS*)-8-(3-Phenoxypropyl)-6*b*,7,8,9,10,10*a*-hexahydro-1*H*-pyrido[3′,4′:4,5]-pyrrolo[1,2,3-*de*]quinoxalin-2(3*H*)-one (**23**)

The synthesis method was analogous to the synthesis of compound **5** in Scheme 1 wherein (3-chloropropoxy)benzene was added in step d instead of 1-(3-chloropropoxy)-4-fluorobenzene. 23% isolated yield. ^1^H NMR (400 MHz, DMSO-*d*_6_): δ 10.33 (s, 1H), 7.30–7.25 (m, 2H), 6.93–6.89 (m, 3H), 6.78 (d, *J* = 7.1 Hz, 1H), 6.64–6.58 (m, 1H), 4.02–3.97 (m, 2H), 3.81 (d, *J* = 14.5 Hz, 1H), 3.33–3.20 (m, 4H), 2.91–2.88 (m, 1H), 2.74–2.62 (m, 1H), 2.44–2.39 (m, 2H), 2.17–2.11 (m, 1H), 1.95–1.79 (m, 3H), and 1.72 (t, *J* = 10.8 Hz, 1H). ^13^C NMR (126 MHz, MeOD-*d*_4_): δ 169.0, 160.3, 139.0, 131.2, 130.5, 125.1, 122.0, 121.8, 119.8, 115.5, 114.1, 67.2, 66.8, 56.9, 56.3, 52.9, 49.7, 42.2, 27.2, and 24.8. HRMS (ESI) *m*/*z* calcd for C_22_H_26_N_3_O_2_ [M + H]^+^, 364.2020; found: 364.2023.

#### (6*bR*,10*aS*)-8-(3-(6-Fluoro-1*H*-indazol-3-yl)propyl)-6*b*,7,8,9,10,10*a*-hexahydro-1*H*-pyrido[3′,4′:4,5]pyrrolo-[1,2,3-*de*]quinoxalin-2(3*H*)-one (**24**)

The synthesis method was analogous to the synthesis of compound **5** in Scheme 1 wherein 3-(3-chloropropyl)-6-fluoro-1*H*-indazole was added in step d instead of 1-(3-chloropropoxy)-4-fluorobenzene. 29% isolated yield. ^1^H NMR (500 MHz, DMSO-*d*_6_): δ 10.36 (s, 1H), 7.77 (dd, *J* = 8.8, 5.3 Hz, 1H), 7.22 (dd, *J* = 9.7, 2.2 Hz, 1H), 6.95 (td, *J* = 9.2, 2.3 Hz, 1H), 6.77 (d, *J* = 7.3 Hz, 1H), 6.64 (t, *J* = 7.5 Hz, 1H), 6.58 (d, *J* = 7.7 Hz, 1H), 3.80 (d, *J* = 14.5 Hz, 1H), 3.30 (d, *J* = 14.4 Hz, 1H), 3.27–3.18 (m, 2H), 2.95–2.88 (m, 2H), 2.84 (dd, *J* = 11.7, 6.4 Hz, 1H), 2.62 (d, *J* = 11.4 Hz, 1H), 2.30 (m, 2H), 2.07 (m, 2H), 1.99–1.85 (m, 3H), 1.82–1.77 (m, 1H), and 1.64 (t, *J* = 11.0 Hz, 1H). ^13^C NMR (126 MHz, DMSO-*d*_6_): δ 166.1, 161.3 (d, *J* = 240.7 Hz), 145.5, 140.9 (d, *J* = 12.6 Hz), 137.5, 130.5, 123.8, 121.7 (d, *J* = 11.3 Hz), 120.0, 118.9, 117.8, 112.2, 108.9 (d, *J* = 25.2 Hz), 95.4 (d, *J* = 25.2 Hz), 65.6, 57.4, 56.3, 51.6, 48.7, 41.1, 26.1, 24.3, and 24.0. HRMS (ESI) *m*/*z* calcd for C_23_H_25_FN_5_O [M + H]^+^, 406.2038; found: 406.2027.

#### (6*bR*,10*aS*)-8-(3-(6-Fluoro-1-methyl-1*H*-indazol-3-yl)propyl)-6*b*,7,8,9,10,10*a*-hexahydro-1*H*-pyrido-[3′,4′:4,5]pyrrolo[1,2,3-*de*]quinoxalin-2(3*H*)-one (**25**)

The synthesis method was analogous to the synthesis of compound **5** in Scheme 1 wherein 3-(3-chloropropyl)-6-fluoro-1-methyl-1*H*-indazole was added in step d instead of 1-(3-chloropropoxy)-4-fluorobenzene. 14% isolated yield. ^1^H NMR (500 MHz, DMSO-*d*_6_): δ 10.36 (s, 1H), 7.76 (dd, *J* = 8.8, 5.2 Hz, 1H), 7.43 (dd, *J* = 10.1, 2.2 Hz, 1H), 6.96 (td, *J* = 9.1, 2.2 Hz, 1H), 6.77 (dd, *J* = 7.4, 1.0 Hz, 1H), 6.63 (t, *J* = 7.5 Hz, 1H), 6.57 (dd, *J* = 1.0 Hz, 1H), 3.92 (s, 3H), 3.80 (d, *J* = 14.5 Hz, 1H), 3.30 (d, *J* = 14.4 Hz, 1H), 3.28–3.16 (m, 2H), 2.93–2.82 (m, 3H), 2.65 (d, *J* = 11.8 Hz, 1H), 2.42–2.25 (m, 2H), 2.11 (t, *J* = 2.8 Hz, 1H), 2.03–1.74 (m, 5H), and 1.68 (t, *J* = 11.0 Hz, 1H). ^13^C NMR (126 MHz, DMSO-*d*_6_): δ 166.1, 161.4 (d, *J* = 240.7 Hz), 144.5, 140.8 (d, *J* = 12.6 Hz), 137.5, 130.2, 123.9, 122.0 (d, *J* = 11.3 Hz), 120.0, 119.3, 117.9, 112.3, 108.9 (d, *J* = 25.2 Hz), 95.1(d, *J* = 26.5 Hz), 65.4, 57.1, 55.8, 51.6, 48.5, 40.7, 35.1, 25.7, and 23.9. HRMS (ESI) *m*/*z* calcd for C_24_H_27_FN_5_O [M + H]^+^, 420.2194; found: 420.2211.

#### (6*bR*,10*aS*)-8-(3-(6-Fluorobenzo[*d*]isoxazol-3-yl)propyl)-6*b*,7,8,9,10,10*a*-hexahydro-1*H*-pyrido[3′,4′:4,5]-pyrrolo[1,2,3-*de*]quinoxalin-2(3*H*)-one (**26**)

The synthesis method was analogous to the synthesis of compound **5** in Scheme 1 wherein 3-(3-chloropropyl)-6-fluorobenzo[*d*]isoxazole was added in step d instead of 1-(3-chloropropoxy)-4-fluorobenzene. 31% isolated yield. ^1^H NMR (500 MHz, DMSO-*d*_6_): δ 10.3 (s, 1H), 8.0–7.9 (m, 1H), 7.7 (dd, *J* = 2.15, 9.19 Hz, 1H), 7.3 (td, *J* = 2.20, 9.09 Hz, 1H), 6.8 (d, *J* = 7.22 Hz, 1H), 6.6 (t, *J* = 7.54 Hz, 1H), 6.6 (d, *J* = 7.75 Hz, 1H), 3.8 (d, *J* = 14.53 Hz, 1H), 3.3 (s, 1H), 3.2 (s, 1H), 3.2–3.1 (m, 1H), 3.0 (t, *J* = 7.45 Hz, 2H), 2.9–2.8 (m, 1H), 2.7–2.5 (m, 1H), 2.4–2.2 (m, 2H), 2.2–2.0 (m, 1H), 2.0–1.8 (m, 3H), and 1.8–1.6 (m, 2H). ^13^C NMR (126 MHz, MeOD-*d*_4_): δ 169.1, 166.0 (d, *J* = 234.4 Hz), 164.9, 159.7, 139.1, 131.7, 124.9, 124.2 (d, *J* = 11.3 Hz), 121.9, 119.7, 119.5, 113.9, 113.5 (d, *J* = 26.5 Hz), 98.0 (d, *J* = 26.5 Hz), 67.5, 58.7, 57.3, 53.0, 49.8, 42.6, 25.5, 25.3, and 23.8. HRMS (ESI) *m*/*z* calcd for C_23_H_24_FN_4_O_2_ [M + H]^+^, 407.1878; found: 407.1881.

#### (6*bR*,10*aS*)-8-(3-(Quinolin-8-yloxy)propyl)-6*b*,7,8,9,10,10*a*-hexahydro-1*H*-pyrido[3′,4′:4,5]pyrrolo[1,2,3-*de*]quinoxalin-2(3*H*)-one (**27**)

The synthesis method was analogous to the synthesis of compound **5** in Scheme 1 wherein 8-(3-chloropropoxy)-quinoline was added in step d instead of 1-(3-chloropropoxy)-4-fluorobenzene. 24% isolated yield. ^1^H NMR (500 MHz, DMSO-*d*_6_): δ 10.1 (s, 1H), 8.9 (dd, *J* = 1.68, 4.25 Hz, 1H), 8.3 (dd, *J* = 1.71, 8.33 Hz, 1H), 7.7–7.5 (m, 3H), 7.3 (dd, *J* = 1.50, 7.44 Hz, 1H), 7.0–6.8 (m, 1H), 6.8–6.5 (m, 2H), 4.4 (t, *J* = 5.85 Hz, 2H), 3.9 (d, *J* = 14.55 Hz, 1H), 3.8–3.6 (m, 2H), 3.5 (s, 1H), 3.4 (d, *J* = 14.47 Hz, 1H), 2.9 (b, 1H), 2.3 (d, *J* = 23.61 Hz, 5H), and 1.3 (d, *J* = 7.00 Hz, 3H). ^13^C NMR (126 MHz, MeOD-*d*_4_): δ 168.8, 155.2, 150.4, 140.9, 139.0, 138.4, 131.2, 129.4, 128.5, 125.5, 123.4, 122.6, 121.9, 120.1, 114.8, 111.6, 69.2, 66.0, 57.1, 56.0, 55.0, 52.9, 40.8, 25.3, and 23.4. HRMS (ESI) *m*/*z* calcd for C_25_H_27_N_4_O_2_ [M + H]^+^, 415.2129; found: 415.2132.

#### (6*bR*,10*aS*)-8-(3-(Benzo[*d*][1,3]dioxol-5-yloxy)propyl)-6*b*,7,8,9,10,10*a*-hexahydro-1*H*-pyrido[3′,4′:4,5]-pyrrolo[1,2,3-*de*]quinoxalin-2(3*H*)-one (**28**)

The synthesis method was analogous to the synthesis of compound **5** in Scheme 1 wherein 5-(3-chloropropoxy)benzo[*d*][1,3]dioxole was added in step d instead of 1-(3-chloropropoxy)-4-fluorobenzene. 22% isolated yield. ^1^H NMR (500 MHz, DMSO-*d*_6_): δ 10.3 (s, 1H), 6.8 (dd, *J* = 6.43, 8.06 Hz, 2H), 6.6 (t, *J* = 7.54 Hz, 1H), 6.6–6.6 (m, 2H), 6.3 (dd, *J* = 2.51, 8.50 Hz, 1H), 5.9 (s, 2H), 3.9 (t, *J* = 6.35 Hz, 2H), 3.8 (d, *J* = 14.54 Hz, 1H), 3.3–3.2 (m, 3H), 2.9–2.8 (m, 1H), 2.7–2.6 (m, 1H), 2.5–2.3 (m, 2H), 2.1 (t, *J* = 11.84 Hz, 1H), 2.0–1.9 (m, 1H), 1.9–1.7 (m, 3H), and 1.7 (t, *J* = 11.01 Hz, 1H). ^13^C NMR (126 MHz, MeOD-*d*_4_): δ 169.1, 155.9, 149.7, 143.1, 139.1, 131.5, 125.0, 122.0, 119.7, 114.0, 108.9, 106.8, 102.4, 98.9, 68.0, 67.4, 57.2, 56.4, 53.0, 49.8, 42.5, 27.4, and 25.1. HRMS (ESI) *m*/*z* calcd for C_23_H_26_N_3_O_4_ [M + H]^+^, 408.1918; found: 408.1917.

#### (6*bR*,10*aS*)-8-(3-(4-Chlorophenoxy)propyl)-6*b*,7,8,9,10,10*a*-hexahydro-1*H*-pyrido[3′,4′:4,5]pyrrolo-[1,2,3-*de*]quinoxalin-2(3*H*)-one (**29**)

The synthesis method was analogous to the synthesis of compound **5** in Scheme 1 wherein 1-chloro-4-(3-chloropropoxy)benzene was added in step d instead of 1-(3-chloropropoxy)-4-fluorobenzene. 21% isolated yield. ^1^H NMR (500 MHz, DMSO-*d*_6_): δ 10.3 (s, 1H), 7.4–7.2 (m, 2H), 6.9 (d, *J* = 8.90 Hz, 2H), 6.8–6.7 (m, 1H), 6.6 (t, *J* = 7.53 Hz, 1H), 6.6 (dd, *J* = 1.04, 7.80 Hz, 1H), 4.0 (t, *J* = 6.37 Hz, 2H), 3.8 (d, *J* = 14.53 Hz, 1H), 3.3–3.2 (m, 3H), 2.9–2.8 (m, 1H), 2.7–2.6 (m, 1H), 2.4 (ddt, *J* = 6.30, 12.61, 19.24 Hz, 2H), 2.1–2.0 (m, 1H), 2.0–1.9 (m, 1H), 1.9–1.7 (m, 3H), and 1.7 (t, *J* = 10.98 Hz, 1H). ^13^C NMR (126 MHz, MeOD-*d*_4_): δ 169.2, 159.2, 139.1, 131.8, 130.3, 126.5, 125.0, 121.9, 119.7, 117.0, 113.9, 67.6, 67.6, 57.5, 56.4, 53.0, 49.9, 42.7, 27.5, and 25.4. HRMS (ESI) *m*/*z* calcd for C_22_H_25_ClN_3_O_2_ [M + H]^+^, 398.1630; found: 398.1628.

#### (6*bR*,10*aS*)-8-(3-(4-Bromophenoxy)propyl)-6*b*,7,8,9,10,10*a*-hexahydro-1*H*-pyrido[3′,4′:4,5]pyrrolo-[1,2,3-*de*]quinoxalin-2(3*H*)-one (**30**)

The synthesis method was analogous to the synthesis of compound **5** in Scheme 1 wherein 1-bromo-4-(3-chloropropoxy)benzene was added in step d instead of 1-(3-chloropropoxy)-4-fluorobenzene. 48% isolated yield. ^1^H NMR (500 MHz, DMSO-*d*_6_): δ 10.35 (s, 1H), 7.66–7.33 (m, 2H), 6.96–6.84 (m, 2H), 6.76 (d, *J* = 7.3, 1.0 Hz, 1H), 6.62 (t, *J* = 7.5 Hz, 1H), 6.59–6.53 (m, 1H), 3.98 (t, *J* = 6.4 Hz, 2H), 3.79 (d, *J* = 14.5 Hz, 1H), 3.29 (d, *J* = 14.5 Hz, 1H), 3.26–3.12 (m, 2H), 2.93–2.80 (m, 1H), 2.73–2.55 (m, 1H), 2.43–2.29 (m, 2H), 2.13–2.03 (m, 1H), 1.96–1.89 (m, 1H), 1.88–1.73 (m, 3H), and 1.65 (t, *J* = 11.0 Hz, 1H). ^13^C NMR (126 MHz, DMSO-*d*_6_): δ 166.1, 157.9, 137.5, 132.1, 130.5, 123.8, 120.0, 117.8, 116.7, 112.2, 111.7, 66.1, 65.6, 56.3, 54.3, 51.6, 48.7, 41.1, 26.2, and 24.3. HRMS (ESI) *m*/*z* calcd for C_22_H_25_BrN_3_O_2_ [M + H]^+^, 442.1125; found: 442.1123.

#### 4-(3-((6*bR*,10*aS*)-2-Oxo-2,3,6*b*,9,10,10*a*-hexahydro-1*H*-pyrido[3′,4′:4,5]pyrrolo[1,2,3-*de*]quinoxalin-8(7*H*)-yl)propoxy)benzonitrile (**31**)

The synthesis method was analogous to the synthesis of compound **5** in Scheme 1 wherein 4-(3-chloropropoxy)benzonitrile was added in step d instead of 1-(3-chloropropoxy)-4-fluorobenzene. 20% isolated yield. ^1^H NMR (500 MHz, DMSO-*d*_6_): δ 10.3 (s, 1H), 7.8 (d, *J* = 8.80 Hz, 2H), 7.1 (d, *J* = 8.79 Hz, 2H), 6.8 (d, *J* = 7.39 Hz, 1H), 6.6 (t, *J* = 7.55 Hz, 1H), 6.6 (d, *J* = 6.78 Hz, 1H), 4.1 (t, *J* = 6.36 Hz, 2H), 3.8 (d, *J* = 14.53 Hz, 1H), 3.3–3.2 (m, 3H), 3.0–2.8 (m, 1H), 2.7–2.6 (m, 1H), 2.5–2.3 (m, 2H), 2.2–2.0 (m, 1H), 2.0–1.8 (m, 3H), 1.8–1.7 (m, 1H), and 1.7 (t, *J* = 11.00 Hz, 1H). ^13^C NMR (126 MHz, MeOD-*d*_4_): δ 169.1, 164.0, 139.1, 135.2, 131.8, 125.0, 121.9, 120.1, 119.7, 116.5, 113.9, 104.7, 67.7, 67.6, 57.5, 56.1, 53.0, 49.8, 42.7, 27.3, and 25.3. HRMS (ESI) *m*/*z* calcd for C_23_H_25_N_4_O_2_ [M + H]^+^, 389.1972; found: 389.1971.

#### (6*bR*,10*aS*)-8-(3-(*p*-Tolyloxy)propyl)-6*b*,7,8,9,10,10*a*-hexahydro-1*H*-pyrido[3′,4′:4,5]pyrrolo[1,2,3-*de*]-quinoxalin-2(3*H*)-one (**32**)

The synthesis method was analogous to the synthesis of compound **5** in Scheme 1 wherein 1-(3-chloropropoxy)-4-methylbenzene was added in step d instead of 1-(3-chloropropoxy)-4-fluorobenzene. 6.9% isolated yield. ^1^H NMR (500 MHz, DMSO-*d*_6_): δ 10.34 (s, 1H), 7.07 (d, *J* = 5 Hz, 2H), 6.84–6.74 (m, 3H), 6.64 (t, *J* = 7.5 Hz, 1H), 6.58 (dd, *J* = 7.8, 1.0 Hz, 1H), 3.95 (t, *J* = 6.4 Hz, 2H), 3.80 (d, *J* = 14.5 Hz, 1H), 3.30–3.24 (m, 2H), 3.22 (dd, *J* = 10.6, 6.4 Hz, 1H), 2.88–2.81 (m, 1H), 2.64 (d, *J* = 11.6 Hz, 1H), 2.38 (dt, *J* = 14.8, 7.1 Hz, 2H), 2.22 (s, 3H), 2.14–2.03 (m, 1H), 1.94 (dd, *J* = 14.4, 2.6 Hz, 1H), 1.90–1.73 (m, 3H), and 1.67 (t, *J* = 11.0 Hz, 1H). ^13^C NMR (126 MHz, MeOD-*d*_4_): δ 169.2, 158.3, 139.1, 131.8, 130.9, 130.8, 125.0, 121.9, 119.7, 115.4, 113.9, 67.6, 67.2, 57.5, 56.5, 53.1, 49.9, 42.7, 27.7, 25.3, and 20.5. HRMS (ESI) *m*/*z* calcd for C_23_H_27_N_3_O_2_ [M + H]^+^, 378.2176; found: 378.2178.

#### (6*bR*,10*aS*)-8-(3-(4-Hydroxyphenoxy)propyl)-6*b*,7,8,9,10,10*a*-hexahydro-1*H*-pyrido[3′,4′:4,5]pyrrolo-[1,2,3-*de*]quinoxalin-2(3*H*)-one (**33**)

The synthesis method was analogous to the synthesis of compound **5** in Scheme 1 wherein 4-(3-chloropropoxy)phenol was added in step d instead of 1-(3-chloropropoxy)-4-fluorobenzene. 6.1% isolated yield. ^1^H NMR (500 MHz, DMSO-*d*_6_): δ 10.33 (s, 1H), 6.80–6.75 (m, 1H), 6.75–6.69 (m, 2H), 6.68–6.60 (m, 3H), 6.57 (dd, *J* = 7.8, 1.0 Hz, 1H), 3.88 (t, *J* = 6.4 Hz, 2H), 3.80 (d, *J* = 14.6 Hz, 1H), 3.27–3.15 (m, 3H), 2.86 (dt, *J* = 10.4, 5.0 Hz, 1H), 2.69–2.59 (m, 1H), 2.46–2.29 (m, 2H), 2.09 (t, *J* = 12.0 Hz, 1H), 1.94 (d, *J* = 13.8 Hz, 1H), 1.81 (p, *J* = 6.4 Hz, 3H), and 1.67 (t, *J* = 10.7 Hz, 1H). ^13^C NMR (126 MHz, DMSO-*d*_6_): δ 166.1, 151.4, 151.1, 137.5, 130.0, 123.9, 120.1, 117.9, 115.7, 115.4, 112.3, 66.1, 65.3, 55.7, 54.3, 51.6, 48.5, 40.6, 26.1, and 23.8. HRMS (ESI) *m*/*z* calcd for C_22_H_26_FN_3_O_3_ [M + H]^+^, 380.1969; found: 380.1961.

#### (6*bR*,10*aS*)-8-(3-(4-Methoxyphenoxy)propyl)-6*b*,7,8,9,10,10*a*-hexahydro-1*H*-pyrido[3′,4′:4,5]pyrrolo-[1,2,3-*de*]quinoxalin-2(3*H*)-one (**34**)

The synthesis method was analogous to the synthesis of compound **5** in Scheme 1 wherein 1-(3-chloropropoxy)-4-methoxybenzene was added in step d instead of 1-(3-chloropropoxy)-4-fluorobenzene. 44% isolated yield. ^1^H NMR (500 MHz, DMSO-*d*_6_): δ 10.3 (s, 1H), 6.8 (d, *J* = 0.58 Hz, 4H), 6.8 (dt, *J* = 0.78, 7.19 Hz, 1H), 6.6 (t, *J* = 7.55 Hz, 1H), 6.6 (dd, *J* = 1.06, 7.74 Hz, 1H), 3.9 (t, *J* = 6.37 Hz, 2H), 3.8 (d, *J* = 14.55 Hz, 1H), 3.7 (s, 3H), 3.3–3.2 (m, 3H), 2.9 (ddd, *J* = 1.64, 5.91, 10.86 Hz, 1H), 2.7 (dd, *J* = 6.12, 9.63 Hz, 1H), 2.5–2.3 (m, 2H), 2.2–2.1 (m, 1H), 2.0–1.9 (m, 1H), 1.9–1.8 (m, 3H), and 1.7 (t, *J* = 10.98 Hz, 1H). ^13^C NMR (126 MHz, MeOD-*d*_4_): δ 169.0, 155.5, 154.3, 139.0, 130.8, 125.2, 122.2, 119.8, 116.5, 115.7, 114.3, 67.4, 66.9, 56.5, 56.2, 56.1, 52.9, 49.6, 41.9, 26.9, and 24.5. HRMS (ESI) *m*/*z* calcd for C_23_H_28_N_3_O_3_ [M + H]^+^, 394.2125; found: 394.2121.

#### (6*bR*,10*aS*)-8-(3-(4-(Benzyloxy)phenoxy)propyl)-6*b*,7,8,9,10,10*a*-hexahydro-1*H*-pyrido[3′,4′:4,5]-pyrrolo[1,2,3-*de*]quinoxalin-2(3*H*)-one (**35**)

The synthesis method was analogous to the synthesis of compound **5** in Scheme 1 wherein 1-(benzyloxy)-4-(3-chloropropoxy)benzene was added in step d instead of 1-(3-chloropropoxy)-4-fluorobenzene. 26% isolated yield. ^1^H NMR (500 MHz, DMSO-*d*_6_): δ 10.34 (s, 1H), 7.46–7.41 (m, 2H), 7.38 (dd, *J* = 8.4, 6.7 Hz, 2H), 7.35–7.29 (m, 1H), 6.96–6.90 (m, 2H), 6.89–6.82 (m, 2H), 6.78 (d, *J* = 7.2 Hz, 1H), 6.64 (t, *J* = 7.5 Hz, 1H), 6.61–6.56 (m, 1H), 5.03 (s, 2H), 3.93 (t, *J* = 6.4 Hz, 2H), 3.81 (d, *J* = 14.5 Hz, 1H), 3.28–3.19 (m, 3H), 2.89 (d, *J* = 8.4 Hz, 1H), 2.64 (t, *J* = 1.9 Hz, 1H), 2.48–2.31 (m, 2H), 2.09 (d, *J* = 10.7 Hz, 1H), 1.95 (d, *J* = 14.5 Hz, 1H), 1.89–1.75 (m, 3H), and 1.69 (d, *J* = 11.6 Hz, 1H). ^13^C NMR (126 MHz, DMSO-*d*_6_): δ 166.1, 152.8, 152.3, 137.5, 137.4, 130.3, 128.4, 127.7, 127.6, 123.9, 120.0, 117.9, 115.7, 115.3, 112.3, 69.6, 66.2, 65.4, 56.0, 54.3, 51.6, 48.6, 40.9, 26.3, and 24.0. HRMS (ESI) *m*/*z* calcd for C_29_H_32_N_3_O_3_ [M + H]^+^, 470.2438; found: 470.2432.

#### (6*bR*,10*aS*)-8-(3-(2-Fluorophenoxy)propyl)-6*b*,7,8,9,10,10*a*-hexahydro-1*H*-pyrido[3′,4′:4,5]pyrrolo[1,2,3-*de*]quinoxalin-2(3*H*)-one (**36**)

The synthesis method was analogous to the synthesis of compound **5** in Scheme 1 wherein 1-(3-chloropropoxy)-2-fluorobenzene was added in step d instead of 1-(3-chloropropoxy)-4-fluorobenzene. 15% isolated yield. ^1^H NMR (500 MHz, DMSO-*d*_6_): δ 10.48 (s, 1H), 7.32–7.09 (m, 3H), 7.04–6.91 (m, 1H), 6.87 (d, *J* = 7.4 Hz, 1H), 6.74 (t, *J* = 7.6 Hz, 1H), 6.68 (dd, *J* = 7.7, 1.1 Hz, 1H), 4.12 (t, *J* = 5.9 Hz, 2H), 3.95 (d, *J* = 14.5 Hz, 1H), 3.69 (dd, *J* = 12.2, 6.3 Hz, 1H), 3.58–3.47 (m, 2H), 3.46–3.36 (m, 2H), 3.30–3.17 (m, 2H), 3.12 (q, *J* = 11.9 Hz, 1H), 2.58 (q, *J* = 11.5 Hz, 1H), 2.42–2.29 (m, 1H), 2.19 (t, *J* = 7.3 Hz, 2H), and 2.11–1.99 (m, 1H). ^13^C NMR (126 MHz, MeOD-*d*_4_): δ 168.7, 154.0 (d, *J* = 244.4 Hz), 147.8 (d, *J* = 10.1 Hz), 138.8, 128.6, 125.8 (d, *J* = 3.8 Hz), 125.5, 122.9 (d, *J* = 6.3 Hz), 122.6, 120.0, 117.1 (d, *J* = 17.6 Hz), 116.3, 114.9, 67.7, 65.5, 61.5, 56.2, 54.6, 52.7, 40.5, 25.4, and 23.0. HRMS (ESI) *m*/*z* calcd for C_22_H_25_FN_3_O_2_ [M + H]^+^, 382.1925; found: 382.1914.

#### (6*bR*,10*aS*)-8-(3-(3-Fluorophenoxy)propyl)-6*b*,7,8,9,10,10*a*-hexahydro-1*H*-pyrido[3′,4′:4,5]pyrrolo[1,2,3-*de*]quinoxalin-2(3*H*)-one (**37**)

The synthesis method was analogous to the synthesis of compound **5** in Scheme 1 wherein 1-(3-chloropropoxy)-3-fluorobenzene was added in step d instead of 1-(3-chloropropoxy)-4-fluorobenzene. 24% isolated yield. ^1^H NMR (500 MHz, CDCl_3_): δ 7.75 (s, 1H), 7.23–7.18 (m, 1H), 6.83 (d, *J* = 7.0 Hz, 1H), 6.73 (t, *J* = 7.5 Hz, 1H), 6.68–6.55 (m, 4H), 4.01 (t, *J* = 5.5 Hz, 2H), 3.96 (d, *J* = 14.5 Hz, 1H), 3.46–3.22 (m, 3H), 3.05–2.87 (m, 1H), 2.83–2.64 (m, 1H), 2.62–2.38 (m, 2H), 2.36–2.13 (m, 1H), 2.05–1.85 (m, 4H), and 1.67–1.49 (m, 1H). ^13^C NMR (126 MHz, MeOD-*d*_4_): δ 169.1, 165.1 (d, *J* = 244.4 Hz), 162.0 (d, *J* = 11.3 Hz), 139.1, 131.9, 131.4 (d, *J* = 10.1 Hz), 125.0, 121.9, 119.7, 113.9, 111.5 (d, *J* = 3.8 Hz), 108.1 (d, *J* = 21.4 Hz), 103.0 (d, *J* = 25.2 Hz), 67.6, 67.6, 57.6, 56.3, 53.0, 49.9, 42.7, 27.5, and 25.4. HRMS (ESI) *m*/*z* calcd for C_22_H_25_FN_3_O_2_ [M + H]^+^, 382.1925; found: 382.1921.

#### 5-Fluoro-2-(3-((6*bR*,10*aS*)-2-oxo-2,3,6*b*,9,10,10*a*-hexahydro-1*H*-pyrido[3′,4′:4,5]pyrrolo[1,2,3-*de*]quinoxalin-8(7*H*)-yl)propoxy)benzamide (**38**)

The synthesis method was analogous to the synthesis of compound **5** in Scheme 1 wherein 2-(3-chloropropoxy)-5-fluorobenzamide was added in step d instead of 1-(3-chloropropoxy)-4-fluorobenzene. 19% isolated yield. ^1^H NMR (500 MHz, DMSO-*d*_6_): δ 10.36 (s, 1H), 7.72 (d, *J* = 18.6 Hz, 2H), 7.53 (dd, *J* = 9.4, 3.4 Hz, 1H), 7.31 (ddd, *J* = 9.1, 7.7, 3.4 Hz, 1H), 7.17 (dd, *J* = 9.2, 4.3 Hz, 1H), 6.82–6.71 (m, 1H), 6.63 (t, *J* = 7.5 Hz, 1H), 6.57 (dd, *J* = 7.8, 1.0 Hz, 1H), 4.14 (t, *J* = 6.2 Hz, 2H), 3.80 (d, *J* = 14.5 Hz, 1H), 3.30 (d, *J* = 14.5 Hz, 1H), 3.27–3.17 (m, 2H), 2.86 (ddd, *J* = 11.4, 6.4, 1.8 Hz, 1H), 2.70–2.60 (m, 1H), 2.45–2.30 (m, 2H), 2.07 (td, *J* = 11.8, 2.8 Hz, 1H), 1.98–1.87 (m, 3H), 1.85–1.73 (m, 1H), and 1.65 (t, *J* = 11.0 Hz, 1H). ^13^C NMR (126 MHz, DMSO-*d*_6_): δ 166.1, 165.0, 155.9 (d, *J* = 236.9 Hz), 152.9, 137.6, 130.5, 124.3 (d, *J* = 6.3 Hz), 123.8, 120.0, 118.7 (d, *J* = 22.7 Hz), 117.8, 116.6 (d, *J* = 23.9 Hz), 114.9 (d, *J* = 7.6 Hz), 112.2, 67.8, 65.6, 56.4, 54.6, 51.6, 48.7, 41.0, 26.0, and 24.2. HRMS (ESI) *m*/*z* calcd for C_23_H_26_FN_4_O_3_ [M + H]^+^, 425.1983; found: 425.1976.

#### (6*bR*,10*aS*)-8-(3-(3,4-Difluorophenoxy)propyl)-6*b*,7,8,9,10,10*a*-hexahydro-1*H*-pyrido[3′,4′:4,5]pyrrolo-[1,2,3-*de*]quinoxalin-2(3*H*)-one (**39**)

The synthesis method was analogous to the synthesis of compound **5** in Scheme 1 wherein 4-(3-chloropropoxy)-1,2-difluorobenzene was added in step d instead of 1-(3-chloropropoxy)-4-fluorobenzene. 47% isolated yield. ^1^H NMR (400 MHz, DMSO-*d*_6_): δ 10.32 (s, 1H), 7.39–7.23 (m, 1H), 7.11–6.99 (m, 1H), 6.82–6.71 (m, 2H), 6.68–6.54 (m, 2H), 4.00 (t, *J* = 6.3 Hz, 2H), 3.80 (d, *J* = 14.5 Hz, 1H), 3.23–3.17 (m, 1H), 2.90–2.81 (m, 1H), 2.70–2.59 (m, 1H), 2.45–2.29 (m, 2H), 2.17–2.05 (m, 1H), 1.99–1.91 (m, 1H), 1.90–1.76 (m, 3H), 1.68 (t, *J* = 10.9 Hz, 1H), and 1.39–1.05 (m, 2H). ^13^C NMR (126 MHz, MeOD-*d*_4_): δ 167.7, 155.6 (d, *J* = 7.6 Hz), 150.3 (dd, *J* = 239.4, 12.6 Hz), 144.7 (dd, *J* = 239.4, 12.6 Hz), 137.7, 130.4, 123.6, 120.5, 118.3, 116.9 (d, *J* = 18.9 Hz), 112.5, 109.9 (dd, *J* = 5.0, 3.8 Hz), 103.6 (d, *J* = 20.2 Hz), 66.7, 66.2, 56.1, 54.9, 51.6, 48.4, 41.3, 26.0, and 23.9. HRMS (ESI) *m*/*z* calcd for C_22_H_24_F_2_N_3_O_2_ [M + H]^+^, 400.1831; found: 400.1826.

#### (6*bR*,10*aS*)-8-(3-(2,4-Difluorophenoxy)propyl)-6*b*,7,8,9,10,10*a*-hexahydro-1*H*-pyrido[3′,4′:4,5]pyrrolo-[1,2,3-*de*]quinoxalin-2(3*H*)-one (**40**)

The synthesis method was analogous to the synthesis of compound **5** in Scheme 1 wherein 1-(3-chloropropoxy)-2,4-difluorobenzene was added in step d instead of 1-(3-chloropropoxy)-4-fluorobenzene. 30% isolated yield. ^1^H NMR (500 MHz, DMSO-*d*_6_): δ 10.47 (s, 1H), 7.46–7.28 (m, 1H), 7.27–7.18 (m, 1H), 7.12–6.97 (m, 1H), 6.94–6.85 (m, 1H), 6.80–6.62 (m, 2H), 4.31–4.06 (m, 2H), 4.02–3.84 (m, 1H), 3.80–3.63 (m, 1H), 3.61–3.34 (m, 3H), 3.28–3.04 (m, 3H), 2.72–2.53 (m, 1H), 2.43–2.29 (m, 1H), 2.28–2.13 (m, 2H), 2.11–2.00 (m, 1H), and 1.28–1.21 (m, 1H). ^13^C NMR (126 MHz, MeOD-*d*_4_): δ 168.7, 158.2 (dd, *J* = 239.4, 8.8 Hz), 153.8 (dd, *J* = 248.2, 12.6 Hz), 144.5 (dd, *J* = 11.3, 3.8 Hz), 138.8, 129.1, 125.5, 122.53, 120.0, 117.3 (dd, *J* = 10.1, 1.3 Hz), 114.8, 111.6 (dd, *J* = 22.7, 3.8 Hz), 105.6 (dd, *J* = 27.7, 22.7 Hz), 68.6, 65.8, 55.8, 54.9, 52.7, 49.5, 40.8, 25.8, and 23.3. HRMS (ESI) *m*/*z* calcd for C_22_H_24_F_2_N_3_O_2_ [M + H]^+^, 400.1831; found: 400.1832.

#### (6*bR*,10*aS*)-8-(3-(4-Fluoro-3-hydroxyphenoxy)propyl)-6*b*,7,8,9,10,10*a*-hexahydro-1*H*-pyrido[3′,4′:4,5]-pyrrolo[1,2,3-*de*]quinoxalin-2(3*H*)-one (**41**)

The synthesis method was analogous to the synthesis of compound **5** in Scheme 1 wherein 5-(3-chloropropoxy)-2-fluorophenol was added in step d instead of 1-(3-chloropropoxy)-4-fluorobenzene. 20% isolated yield. ^1^H NMR (500 MHz, DMSO-*d*_6_): δ 10.34 (s, 1H), 9.83 (s, 1H), 6.99 (dd, *J* = 11.1, 8.9 Hz, 1H), 6.77 (d, *J* = 7.3 Hz, 1H), 6.63 (t, *J* = 7.5 Hz, 1H), 6.58 (d, *J* = 7.7 Hz, 1H), 6.47 (dd, *J* = 7.5, 3.0 Hz, 1H), 6.29 (dt, *J* = 8.9, 3.1 Hz, 1H), 3.90 (t, *J* = 6.4 Hz, 2H), 3.80 (d, *J* = 14.5 Hz, 1H), 3.40–3.11 (m, 3H), 2.92–2.78 (m, 1H), 2.63 (d, *J* = 11.1 Hz, 1H), 2.44–2.26 (m, 2H), 2.08 (ddd, *J* = 11.8, 9.5, 2.7 Hz, 1H), 1.94 (dd, *J* = 14.5, 2.7 Hz, 1H), 1.82 (p, *J* = 8.4, 7.5 Hz, 3H), and 1.66 (t, *J* = 11.0 Hz, 1H). ^13^C NMR (126 MHz, MeOD-*d*_4_): δ 169.2, 157.0, 147.9 (d, *J* = 231.8 Hz), 146.7 (d, *J* = 15.1 Hz), 139.1, 131.9, 125.0, 121.9, 119.7, 116.6 (d, *J* = 20.2 Hz), 113.9, 105.8 (d, *J* = 6.3 Hz), 105.4, 67.7, 57.6, 56.4, 53.1, 49.9, 42.7, 27.6, and 25.3. HRMS (ESI) *m*/*z* calcd for C_22_H_25_FN_3_O_3_ [M + H]^+^, 398.1874; found, 398.1868.

#### (6*bR*,10*aS*)-8-(3-(4-Fluoro-2-hydroxyphenoxy)propyl)-6*b*,7,8,9,10,10*a*-hexahydro-1*H*-pyrido[3′,4′:4,5]-pyrrolo[1,2,3-*de*]quinoxalin-2(3*H*)-one (**42**)

The synthesis method was analogous to the synthesis of compound **5** in Scheme 1 wherein 2-(3-chloropropoxy)-5-fluorophenol was added in step d instead of 1-(3-chloropropoxy)-4-fluorobenzene. 5.3% isolated yield. ^1^H NMR (500 MHz, DMSO-*d*_6_): δ 11.53 (s, 1H), 10.34 (s, 1H), 6.92 (dd, *J* = 8.8, 5.8 Hz, 1H), 6.78 (dd, *J* = 7.3, 1.0 Hz, 1H), 6.67–6.56 (m, 3H), 6.50 (td, *J* = 8.6, 3.1 Hz, 1H), 4.01–3.86 (m, 2H), 3.81 (d, *J* = 14.5 Hz, 1H), 3.31–3.19 (m, 3H), 3.00–2.88 (m, 1H), 2.75 (d, *J* = 11.7 Hz, 1H), 2.45 (dd, *J* = 12.4, 6.1 Hz, 2H), 2.18–2.05 (m, 1H), 2.01–1.92 (m, 1H), 1.92–1.77 (m, 3H), and 1.69 (t, *J* = 10.8 Hz, 1H). ^13^C NMR (126 MHz, DMSO-*d*_6_): δ 166.1, 155.3 (d, *J* = 234.4 Hz), 147.4 (d, *J* = 10.1 Hz), 143.6, 137.5, 130.4, 123.9, 120.0, 117.8, 115.4 (d, *J* = 8.8 Hz), 112.2, 106.5 (d, *J* = 22.7 Hz), 102.4 (d, *J* = 26.5 Hz), 67.7, 65.6, 56.3, 54.5, 51.6, 48.6, 40.9, 26.0, and 24.1. HRMS (ESI) *m*/*z* calcd for C_22_H_25_FN_3_O_3_ [M + H]^+^, 398.1874; found: 398.1862.

#### (6*bR*,10*aS*)-8-(3-(4-Fluoro-2,6-dimethylphenoxy)propyl)-6*b*,7,8,9,10,10*a*-hexahydro-1*H*-pyrido[3′,4′:4,5]-pyrrolo[1,2,3-*de*]quinoxalin-2(3*H*)-one (**43**)

The synthesis method was analogous to the synthesis of compound **5** in Scheme 1 wherein 2-(3-chloropropoxy)-5-fluoro-1,3-dimethylbenzene was added in step d instead of 1-(3-chloropropoxy)-4-fluorobenzene. 66% isolated yield. ^1^H NMR (400 MHz, DMSO-*d*_6_): δ 10.61 (s, 1H), 7.13 (d, *J* = 9.2 Hz, 2H), 7.05 (dd, *J* = 7.3, 1.1 Hz, 1H), 6.92 (t, *J* = 10 Hz, 1H), 6.87 (dd, *J* = 7.8, 1.1 Hz, 1H), 4.09 (d, *J* = 14.5 Hz, 1H), 4.02 (t, *J* = 6.2 Hz, 2H), 3.58 (s, 3H), 3.53–3.43 (m, 1H), 3.21–3.10 (m, 1H), 2.98–2.87 (m, 1H), 2.81–2.76 (m, 1H), 2.76–2.65 (m, 2H), 2.50 (s, 6H), 2.45–2.36 (m, 1H), 2.28–2.20 (m, 1H), 2.20–2.11 (m, 1H), and 2.00 (t, *J* = 11.0 Hz, 1H). ^13^C NMR (126 MHz, DMSO-*d*_6_): δ 166.1, 157.6 (d, *J* = 239.4 Hz), 151.7, 137.5, 132.5 (d, *J* = 8.8 Hz), 130.4, 123.8, 120.0, 117.8, 114.5 (d, *J* = 22.7 Hz), 112.2, 69.9, 65.6, 56.3, 54.2, 51.6, 48.7, 41.2, 27.2, 24.3, and 15.9. HRMS (ESI) *m*/*z* calcd for C_24_H_29_FN_3_O_2_ [M + H]^+^, 410.2238; found: 410.2225.

#### (6*bR*,10*aS*)-8-(4-(4-Fluorophenyl)butyl)-6*b*,7,8,9,10,10*a*-hexahydro-1*H*-pyrido[3′,4′:4,5]pyrrolo-[1,2,3-*de*]quinoxalin-2(3*H*)-one (**44**)

The synthesis method was analogous to Scheme 1 for the synthesis of compound **5** wherein 1-(4-chlorobutyl)-4-fluorobenzene was added in step d instead of 1-(3-chloropropoxy)-4-fluorobenzene. 38% yield. ^1^H NMR (400 MHz, DMSO-*d*_6_): δ 10.32 (s, 1H), 7.22 (dd, *J* = 8.5, 5.8 Hz, 2H), 7.12–7.03 (m, 2H), 6.76 (dd, *J* = 7.3, 1.1 Hz, 1H), 6.64 (t, *J* = 10 Hz, 1H), 6.58 (dd, *J* = 7.8, 1.1 Hz, 1H), 3.80 (d, *J* = 14.5 Hz, 1H), 3.29–3.23 (m, 2H), 3.22–3.16 (m, 1H), 2.88–2.79 (m, 1H), 2.62 (d, *J* = 11.7 Hz, 1H), 2.57 (t, *J* = 7.5 Hz, 2H), 2.36–2.20 (m, 2H), 2.17–2.04 (m, 1H), 1.99–1.87 (m, 1H), 1.86–1.73 (m, 1H), 1.68 (t, *J* = 11.0 Hz, 1H), 1.61–1.49 (m, 2H), and 1.50–1.36 (m, 2H). ^13^C NMR (126 MHz, MeOD-*d*_4_): δ 169.3, 162.8 (d, *J* = 243.2 Hz), 138.8, 138.8 (d, *J* = 2.5 Hz), 131.0 (d, *J* = 7.6 Hz), 129.2, 125.5, 122.5, 119.9, 116.0 (d, *J* = 21.4 Hz), 114.8, 65.9, 57.9, 54.6, 52.7, 40.6, 35.3, 29.6, 24.8, and 23.0. HRMS (ESI) *m*/*z* calcd for C_23_H_27_FN_3_O [M + H]^+^, 380.2133; found: 380.2120.

#### (6*bR*,10*aS*)-8-(3-((4-Fluorophenyl)thio)propyl)-6*b*,7,8,9,10,10*a*-hexahydro-1*H*-pyrido[3′,4′:4,5]pyrrolo-[1,2,3-*de*]quinoxalin-2(3*H*)-one (**45**)

The synthesis method was analogous to the synthesis of compound **5** in Scheme 1 wherein (3-chloropropyl)(4-fluorophenyl)sulfane was added in step d instead of 1-(3-chloropropoxy)-4-fluorobenzene. 39% isolated yield. ^1^H NMR (500 MHz, DMSO-*d*_6_): δ 10.35 (s, 1H), 6.97–6.84 (m, 2H), 6.78 (d, *J* = 7.2 Hz, 1H), 6.71–6.61 (m, 1H), 6.61–6.56 (m, 1H), 6.56–6.46 (m, 2H), 5.51 (t, *J* = 5.7 Hz, 1H), 3.81 (d, *J* = 14.6 Hz, 1H), 3.31–3.25 (m, 1H), 3.25–3.18 (m, 1H), 3.00 (q, *J* = 6.5 Hz, 2H), 2.89–2.83 (m, 1H), 2.68–2.59 (m, 1H), 2.39–2.28 (m, 2H), 2.14–2.04 (m, 1H), 1.99–1.92 (m, 1H), 1.85–1.76 (m, 1H), and 1.71–1.63 (m, 3H). ^13^C NMR (126 MHz, MeOD-*d*_4_): δ 169.1, 163.2 (d, *J* = 245.7 Hz), 139.1, 133.5 (d, *J* = 8.8 Hz), 132.9, 131.8, 125.0, 121.9, 119.7, 116.9 (d, *J* = 22.7 Hz), 113.9, 67.6, 58.2, 57.5, 53.0, 49.9, 42.7, 33.5, 27.2, and 25.3. HRMS (ESI) *m*/*z* calcd for C_22_H_25_FN_3_OS [M + H]^+^, 398.1697; found: 398.1700.

#### (6*bR*,10*aS*)-8-(3-((4-Fluorophenyl)sulfonyl)propyl)-6*b*,7,8,9,10,10*a*-hexahydro-1*H*-pyrido[3′,4′:4,5]-pyrrolo-[1,2,3-*de*]quinoxalin-2(3*H*)-one (**46**)

The synthesis method was analogous to the synthesis of compound **5** in Scheme 1 wherein 1-((3-chloropropyl)sulfonyl)-4-fluorobenzene was added in step d instead of 1-(3-chloropropoxy)-4-fluorobenzene. 34% isolated yield. ^1^H NMR (400 MHz, DMSO-*d*_6_): δ 10.31 (s, 1H), 8.04–7.93 (m, 2H), 7.52 (dd, *J* = 8.8 Hz, 2H), 6.73 (dd, *J* = 7.4, 1.1 Hz, 1H), 6.63 (dd, *J* = 7.5 Hz, 1H), 6.57 (dd, *J* = 7.8, 1.1 Hz, 1H), 3.78 (d, *J* = 14.5 Hz, 1H), 3.37–3.29 (m, 3H), 3.27 (s, 1H), 3.20–3.11 (m, 1H), 2.72–2.63 (m, 1H), 2.46 (s, 1H), 2.36–2.20 (m, 2H), 2.13–2.01 (m, 1H), 1.96–1.84 (m, 1H), and 1.82–1.58 (m, 4H). ^13^C NMR (126 MHz, DMSO-*d*_6_): δ 166.1, 165.0 (d, *J* = 253.3 Hz), 137.5, 135.4 (d, *J* = 2.5 Hz), 130.9 (d, *J* = 10.08 Hz), 130.3, 123.8, 120.0, 117.7, 116.7(d, *J* = 22.7 Hz), 112.2, 65.4, 55.8, 55.2, 52.8, 51.5, 48.4, 41.0, 24.2, and 20.0. HRMS (ESI) *m*/*z* calcd for C_22_H_25_FN_3_O_3_S [M + H]^+^, 430.1595; found: 430.1593.

#### (6*bR*,10*aS*)-8-(3-((4-Fluorophenyl)amino)propyl)-6*b*,7,8,9,10,10*a*-hexahydro-1*H*-pyrido[3′,4′:4,5]-pyrrolo-[1,2,3-*de*]quinoxalin-2(3*H*)-one (**47**)

The synthesis method was analogous to the synthesis of compound **5** in Scheme 1 wherein *N*-(3-chloropropyl)-4-fluoroaniline was added in step d instead of 1-(3-chloropropoxy)-4-fluorobenzene. 7% isolated yield. ^1^H NMR (500 MHz, DMSO-*d*_6_): δ 10.35 (s, 1H), 6.97–6.84 (m, 2H), 6.78 (d, *J* = 7.2 Hz, 1H), 6.71–6.61 (m, 1H), 6.61–6.56 (m, 1H), 6.56–6.46 (m, 2H), 5.51 (t, *J* = 5.7 Hz, 1H), 3.81 (d, *J* = 14.6 Hz, 1H), 3.31–3.25 (m, 2H), 3.25–3.18 (m, 1H), 3.00 (q, *J* = 6.5 Hz, 2H), 2.89–2.83 (m, 1H), 2.68–2.59 (m, 1H), 2.39–2.28 (m, 2H), 2.14–2.04 (m, 1H), 1.99–1.92 (m, 1H), 1.85–1.76 (m, 1H), and 1.71–1.63 (m, 3H). ^13^C NMR (126 MHz, MeOD-*d*_4_): δ 169.2, 157.1 (d, *J* = 233.1 Hz), 146.8, 139.1, 131.9, 125.0, 121.9, 119.7, 116.2 (d, *J* = 21.4 Hz), 115.0 (d, *J* = 7.6 Hz), 113.9, 67.6, 57.7, 57.5, 53.0, 49.9, 44.1, 42.8, 27.1, and 25.4. HRMS (ESI) *m*/*z* calcd for C_22_H_26_FN_4_O [M + H]^+^, 381.2085; found: 381.2078.

#### (6*bR*,10*aS*)-8-(3-((4-Fluorophenyl)(methyl)amino)propyl)-6*b*,7,8,9,10,10*a*-hexahydro-1*H*-pyrido[3′,4′:4,5]pyrrolo[1,2,3-*de*]quinoxalin-2(3*H*)-one (**48**)

The synthesis method was analogous to the synthesis of compound **5** in Scheme 1 wherein *N*-(3-chloropropyl)-4-fluoro-*N*-methylaniline was added in step d instead of 1-(3-chloropropoxy)-4-fluorobenzene. 24% isolated yield. ^1^H NMR (500 MHz, DMSO-*d*_6_): δ 10.35 (s, 1H), 7.01–6.97 (m, 2H), 6.78–6.75 (m, 1H), 6.72–6.68 (m, 2H), 6.64 (dd, *J* = 7.5 Hz, 1H), 6.60–6.56 (m, 1H), 3.81 (d, *J* = 14.5 Hz, 1H), 3.39–3.32 (m, 2H), 3.30–3.26 (m, 2H), 3.25–3.19 (m, 1H), 2.84 (s, 3H), 2.83–2.76 (m, 1H), 2.63–2.56 (m, 1H), 2.30–2.19 (m, 2H), 2.11–2.03 (m, 1H), 1.98–1.92 (m, 1H), 1.85–1.76 (m, 1H), and 1.68–1.59 (m, 3H). ^13^C NMR (126 MHz, DMSO-*d*_6_): δ 166.1, 154.2 (d, *J* = 233.1 Hz), 146.2, 137.5, 130.5, 123.8, 120.0, 117.8, 115.2(d, *J* = 21.4 Hz), 113.1 (d, *J* = 7.6 Hz), 112.2, 65.6, 56.3, 55.0, 51.6, 50.2, 48.7, 41.2, 38.2, 24.3, and 23.3. HRMS (ESI) *m*/*z* calcd for C_23_H_28_FN_4_O [M + H]^+^, 395.2242; found: 395.2240.

#### (6*bR*,10*aS*)-8-(2-(4-Fluorophenoxy)ethyl)-6*b*,7,8,9,10,10*a*-hexahydro-1*H*-pyrido[3′,4′:4,5]pyrrolo[1,2,3-*de*]quinoxalin-2(3*H*)-one (**49**)

The synthesis method was analogous to the synthesis of compound **5** in Scheme 1 wherein 1-(2-chloroethoxy)-4-fluorobenzene was added in step d instead of 1-(3-chloropropoxy)-4-fluorobenzene. 34% isolated yield. ^1^H NMR (500 MHz, DMSO-*d*_6_): δ 10.34 (s, 1H), 7.13–7.06 (m, 2H), 6.98–6.90 (m, 2H), 6.77 (dd, *J* = 7.4, 1.0 Hz, 1H), 6.64 (t, *J* = 7.5 Hz, 1H), 6.58 (dd, *J* = 7.9, 1.0 Hz, 1H), 4.04 (t, *J* = 5.8 Hz, 2H), 3.81 (d, *J* = 14.5 Hz, 1H), 3.23 (m, 3H), 2.98–2.90 (m, 1H), 2.78–2.69 (m, 1H), 2.70–2.57 (m, 2H), 2.23 (td, *J* = 12.0, 2.8 Hz, 1H), 1.94 (dq, *J* = 14.4, 2.5 Hz, 1H), and 1.87–1.73 (m, 2H). ^13^C NMR (126 MHz, DMSO-*d*_6_): δ 166.1, 156.4 (d, *J* = 236.9 Hz), 154.8, 137.6, 130.4, 123.9, 120.0, 117.8, 115.8 (d, *J* = 13.9 Hz), 115.7, 112.2, 66.1, 65.4, 56.7, 56.6, 51.7, 48.9, 41.0, and 24.2. HRMS (ESI) *m*/*z* calcd for C_21_H_23_FN_3_O_2_ [M + H]^+^, 368.1769; found: 368.1759.

#### (6*bR*,10*aS*)-8-(4-(4-Fluorophenoxy)butyl)-6*b*,7,8,9,10,10*a*-hexahydro-1*H*-pyrido[3′,4′:4,5]pyrrolo[1,2,3-*de*]quinoxalin-2(3*H*)-one (**50**)

The synthesis method was analogous to the synthesis of compound **5** in Scheme 1 wherein 1-(4-chlorobutoxy)-4-fluorobenzene was added in step d instead of 1-(3-chloropropoxy)-4-fluorobenzene. 31% isolated yield. ^1^H NMR (500 MHz, DMSO-*d*_6_): δ 10.33 (s, 1H), 7.14–7.04 (m, 2H), 6.97–6.89 (m, 2H), 6.79–6.71 (m, 1H), 6.63 (t, *J* = 7.6 Hz, 1H), 6.57 (dd, *J* = 7.9, 1.0 Hz, 1H), 3.95 (t, *J* = 6.5 Hz, 2H), 3.79 (d, *J* = 14.5 Hz, 1H), 3.29–3.22 (m, 2H), 3.18 (dt, *J* = 10.7, 6.5 Hz, 1H), 2.90–2.75 (m, 1H), 2.68–2.57 (m, 1H), 2.40–2.17 (m, 2H), 2.12–2.00 (m, 1H), 1.92 (dd, *J* = 14.5, 2.6 Hz, 1H), 1.86–1.73 (m, 1H), 1.74–1.60 (m, 3H), and 1.61–1.48 (m, 2H). ^13^C NMR (126 MHz, MeOD-*d*_4_): δ 169.1, 159.6 (d, *J* = 236.9 Hz), 156.7, 139.1, 131.9, 125.0, 121.9, 119.7, 116.7, 116.6 (d, *J* = 13.9 Hz), 113.9, 69.3, 67.6, 59.4, 57.5, 53.0, 49.8, 42.7, 28.4, 25.3, and 24.2. HRMS (ESI) *m*/*z* calcd for C_23_H_27_FN_3_O_2_ [M + H]^+^, 396.2082; found: 396.2068.

#### (6*bR*,10*aS*)-8-(3-(4-Fluorophenoxy)propyl)-1-methyl-6*b*,7,8,9,10,10*a*-hexahydro-1*H*-pyrido[3′,4′:4,5]-pyrrolo[1,2,3-*de*]quinoxalin-2(3*H*)-one (**51**)

The synthesis method was analogous to the synthesis of compound **5** in Scheme 1 wherein 2-chloropropanamide was added in step a instead of 2-chloroacetamide. 17% isolated yield. ^1^H NMR (500 MHz, DMSO-*d*_6_): δ 10.85 (s, 1H), 7.62–7.46 (m, 2H), 7.43–7.31 (m, 2H), 7.26 (s, 1H), 7.15 (s, 1H), 7.06 (s, 1H), 4.43 (t, *J* = 6.2 Hz, 2H), 4.28–3.83 (m, 3H), 3.74 (s, 1H), 3.68–3.36 (m, 2H), 3.16 (d, *J* = 0.7 Hz, 1H), 2.93 (p, *J* = 1.9 Hz, 2H), 2.88–2.73 (m, 2H), 2.67–2.55 (m, 2H), and 1.91 (d, *J* = 6.6 Hz, 3H). ^13^C NMR (126 MHz, MeOD-*d*_4_): δ 170.9, 158.7(d, *J* = 238.1 Hz), 156.2, 139.9, 129.2, 125.8, 122.8, 119.4, 116.8 (d, *J* = 2.52 Hz), 116.7 (d, *J* = 12.6 Hz), 114.5, 66.9, 66.5, 60.8, 55.9, 55.1, 49.2, 41.6, 26.9, 25.8, and 17.3. HRMS (ESI) *m*/*z* calcd for C_23_H_27_FN_3_O_2_ [M + H]^+^, 396.2082; found: 396.2081.

#### (6*bR*,10*aS*)-8-(3-(4-Fluorophenoxy)propyl)-1,1-dimethyl-6*b*,7,8,9,10,10*a*-hexahydro-1*H*-pyrido[3′,4′:4,5]-pyrrolo[1,2,3-*de*]quinoxalin-2(3*H*)-one (**52**)

##### Step 1

DMF (25 mL) was added to a Schlenk tube (50 mL) containing ethyl (6*bR*,10*aS*)-2-oxo-2,3,6*b*,9,10,10*a*-hexahydro-1*H*-pyrido[3′,4′:4,5]-pyrrolo[1,2,3-*de*]quinoxaline-8(7*H*)-carboxylate (2.0 g, 6.64 mmol), potassium iodide (1.43 g, 8.63 mmol), potassium carbonate (2.29 g, 16.6 mmol), DMAP (0.81 g, 6.64 mmol), DIPEA (1.73 mL, 9.96 mmol), and 4-methoxybenzyl bromide (2.24 mL, 16.6 mmol). The mixture was heated to 100 °C and stirred for 2 days. The solvent was evaporated, and the residue was purified by column chromatography to give the intermediate compound ethyl (6*bR*,10*aS*)-3-(4-methoxybenzyl)-2-oxo-2,3,6*b*,9,10,10*a*-hexahydro-1*H*-pyrido-[3′,4′:4,5]pyrrolo[1,2,3-*de*]quinoxaline-8(7*H*)-carboxylate (0.43 g, 15% yield). MS (ESI) *m*/*z*: 422.2 [M + H]^+^.

##### Step 2

THF (3 mL) was added to a Schlenk tube (25 mL) containing ethyl (6*bR*,10*aS*)-3-(4-methoxybenzyl)-2-oxo-2,3,6*b*,9,10,10*a*-hexahydro-1*H*-pyrido[3′,4′:4,5]pyrrolo[1,2,3-*de*]quinoxaline-8(7*H*)-carboxylate (0.43 g, 1.0 mmol). The mixture was cooled to −78 °C, and lithium bis(trimethylsilyl)amide solution (1 M in toluene, 1.5 mL) was added slowly. After completion, the mixture was stirred at −78 °C for 1.5 h, and iodomethane (0.19 mL, 3.0 mmol) was added. After another 1 h stirring at −78 °C, the reaction was warmed up to 0 °C and stirred for 1 h. The mixture was quenched with saturated sodium chloride and extracted with ethyl acetate (3 × 30 mL). The combined organic phase was concentrated, and the residue was purified by column chromatography to give the intermediate compound ethyl (6*bR*,10*aS*)-3-(4-methoxybenzyl)-1-methyl-2-oxo-2,3,6*b*,9,10,10*a*-hexahydro-1*H*-pyrido[3′,4′:4,5]pyrrolo[1,2,3-*de*]quinoxaline-8(7*H*)-carboxylate as a white solid (0.38 g, 82% yield). MS (ESI) *m*/*z*: 436.2 [M + H]^+^.

##### Step 3

THF (10 mL) was added to a Schlenk tube (50 mL) containing ethyl (6*bR*,10*aS*)-3-(4-methoxybenzyl)-1-methyl-2-oxo-2,3,6*b*,9,10,10*a*-hexahydro-1*H*-pyrido[3′,4′:4,5]pyrrolo[1,2,3-*de*]quinoxaline-8(7*H*)-carboxylate (1.01 g, 2.32 mmol). The mixture was cooled to −78 °C, and LDA (4.64 mL, 9.28 mmol) was added slowly. The mixture was stirred at −78 °C for 1 h, and iodomethane (0.58 mL, 9.28 mmol) was added. The resulting mixture was stirred at room temperature for 2 days and quenched with water. The mixture was concentrated, and the obtained residue was purified by column chromatography to afford the intermediate compound ethyl (6*bR*,10*aS*)-3-(4-methoxybenzyl)-1,1-dimethyl-2-oxo-2,3,6*b*,9,10,10*a*-hexahydro-1*H*-pyrido[3′,4′:4,5]pyrrolo[1,2,3-*de*]quinoxaline-8(7*H*)-carboxylate (0.26 g, 25% yield). MS (ESI) *m*/*z*: 450.2 [M + H]^+^.

##### Step 4

Trifluoromethanesulfonic acid (0.47 mL, 5.32 mmol) was added to a Schlenk tube (25 mL) containing ethyl (6*bR*,10*aS*)-3-(4-methoxybenzyl)-1,1-dimethyl-2-oxo-2,3,6*b*,9,10,10*a*-hexahydro-1*H*-pyrido[3′,4′:4,5]pyrrolo-[1,2,3-*de*]quinoxaline-8(7*H*)-carboxylate (0.6 g, 1.33 mmol) and trifluoroacetic acid (1.0 mL, 13.3 mmol) at room temperature. After stirred at room temperature for 1 h, the mixture was concentrated and neutralized with 7 N ammonium in methanol. The obtained residue was purified by column chromatography to give the intermediate compound ethyl (6*bR*,10*aS*)-1,1-dimethyl-2-oxo-2,3,6*b*,9,10,10*a*-hexahydro-1*H*-pyrido-[3′,4′:4,5]-pyrrolo-1,2,3-*de*]quinoxaline-8(7*H*)-carboxylate (0.3 g, 68% yield). MS (ESI) *m*/*z*: 330.1 [M + H]^+^.

##### Step 5

Hydrogen bromide solution (33 wt % in acetic acid, 2.4 mL, 13 mmol) was added to a Schlenk tube (25 mL) containing ethyl (6*bR*,10*aS*)-1,1-dimethyl-2-oxo-2,3,6*b*,9,10,10*a*-hexahydro-1*H*-pyrido[3′,4′:4,5]pyrrolo-[1,2,3-*de*]quinoxaline-8(7*H*)-carboxylate (0.33 g, 1.0 mmol). The resulting mixture was heated to 50 °C and stirred for 13 h. The reaction mixture was quenched with ethyl acetate (12 mL) and filtered. The filtered cake was collected and neutralized with 7 N ammonium in methanol. The neutralized solution was evaporated to dryness to afford the intermediate compound (6*bR*,10*aS*)-1,1-dimethyl-6*b*,7,8,9,10,10*a*-hexahydro-1*H*-pyrido[3′,4′:4,5]pyrrolo[1,2,3-*de*]quinoxalin-2(3*H*)-one (0.302 g). This crude intermediate was directly used in the next reaction without further purification. ^1^H NMR (500 MHz, deuterium oxide): δ 8.36 (s, 1H), 7.43–7.16 (m, 4H), 7.16–7.02 (m, 2H), 5.34–4.96 (m, 2H), 4.39–4.06 (m, 2H), 3.79–3.45 (m, 4H), 3.37–3.12 (m, 2H), 3.15–2.98 (m, 2H), 2.62–2.37 (m, 2H), and 2.31–2.14 (m, 2H). HRMS (ESI) *m*/*z* calcd for C_15_H_20_N_3_O [M + H]^+^, 258.1601; found: 258.1590.

##### Step 6

The synthesis method in step 6 was analogous to the synthesis of compound **5** in Scheme 1 wherein (6*bR*,10*aS*)-1,1-dimethyl-6*b*,7,8,9,10,10*a*-hexahydro-1*H*-pyrido[3′,4′:4,5]pyrrole-[1,2,3-*de*]quinoxalin-2(3*H*)-one was added in step d instead of (6*bR*,10*aS*)-6*b*,7,8,9,10,10*a*-hexahydro-1*H*-pyrido[3′,4′:4,5]-pyrrolo[1,2,3-*de*]quinoxalin-2(3*H*)-one. The title compound (**52**) was isolated in a 16% yield. ^1^H NMR (500 MHz, DMSO-*d*_6_): δ 10.27 (s, 1H), 7.13–7.06 (m, 2H), 6.95–6.89 (m, 2H), 6.74 (d, *J* = 7.3 Hz, 1H), 6.62 (dd, *J* = 7.6 Hz, 1H), 6.57–6.53 (m, 1H), 3.95 (t, *J* = 6.4 Hz, 2H), 3.88–3.78 (m, 1H), 3.30–3.20 (m, 1H), 2.88–2.78 (m, 1H), 2.72–2.59 (m, 1H), 2.44–2.31 (m, 2H), 2.27–2.09 (m, 2H), 1.96–1.79 (m, 3H), 1.73 (t, *J* = 11.0 Hz, 1H), 1.54 (s, 3H), and 1.07 (s, 3H). ^13^C NMR (126 MHz, MeOD-*d*_4_): δ 173.4, 158.6 (d, *J* = 236.9 Hz), 156.7, 138.8, 131.7, 124.6, 121.5, 119.2, 116.7, 116.6 (d, *J* = 15.1 Hz), 113.3, 67.8, 62.3, 60.4, 57.6, 56.5, 49.6, 43.3, 28.9, 27.6, 25.6, and 18.3. HRMS (ESI) *m*/*z* calcd for C_24_H_29_FN_3_O_2_ [M + H]^+^, 410.2238; found: 410.2238.

#### (8*aS*,12*aR*)-11-(3-(4-Fluorophenoxy)propyl)-6,7,8*a*,9,10,11,12,12*a*-octahydro-[1,4]diazepino[3,2,1-*hi*]pyrido[4,3-*b*]indol-5(4*H*)-one (**53**)

The synthesis method was analogous to the synthesis of compound **5** in Scheme 1 wherein 3-chloropropanamide was added in step a, instead of 2-chloroacetamide. 28% isolated yield. ^1^H NMR (500 MHz, CDCl_3_): δ 7.33 (s, 1H), 7.02–6.92 (m, 2H), 6.88 (d, *J* = 7.2 Hz, 1H), 6.78 (dd, *J* = 9.3, 4.0 Hz, 3H), 6.63 (d, *J* = 8.0 Hz, 1H), 4.01 (t, *J* = 5.9 Hz, 2H), 3.57 (s, 1H), 3.40 (ddd, *J* = 12.7, 7.5, 2.5 Hz, 1H), 3.16 (ddd, *J* = 12.7, 9.0, 1.9 Hz, 1H), 2.99–2.92 (m, 2H), 2.92–2.84 (m, 2H), 2.26 (d, *J* = 75.0 Hz, 4H), and 1.56 (b, 5H). ^13^C NMR (126 MHz, MeOD-*d*_4_): δ 175.2, 158.8 (d, *J* = 238.1 Hz), 156.3, 141.4, 133.6, 123.8, 121.3, 120.9, 120.5, 116.8, 116.6, 67.1, 67.0, 63.8, 56.0, 55.1, 44.5, 40.2, 38.1, 26.3, and 24.2. HRMS (ESI) *m*/*z* calcd for C_23_H_27_FN_3_O_2_ [M + H]^+^, 396.2082; found: 396.2079.

#### (6*bS*,10*aR*)-8-(3-(4-Fluorophenoxy)propyl)-6*b*,7,8,9,10,10*a*-hexahydro-1*H*-pyrido[3′,4′:4,5]pyrrolo[1,2,3-*de*]quinoxalin-2(3*H*)-one (**54**)

The synthesis method was analogous to the synthesis of compound **5** in Scheme 1 wherein ethyl (4*aR*,9*bS*)-6-bromo-1,3,4,4*a*,5,9*b*-hexahydro-2*H*-pyrido[4,3-*b*]indole-2-carboxylate was added in step a, instead of ethyl (4*aS*,9*bR*)-6-bromo-1,3,4,4*a*,5,9*b*-hexahydro-2*H*-pyrido[4,3-*b*]indole-2-carboxylate. 47% isolated yield. ^1^H NMR (500 MHz, DMSO-*d*_6_): δ 10.34 (s, 1H), 7.10 (t, *J* = 8.8 Hz, 2H), 6.97–6.88 (m, 2H), 6.77 (d, *J* = 7.3 Hz, 1H), 6.64 (t, *J* = 7.6 Hz, 1H), 6.58 (dd, *J* = 7.8, 1.0 Hz, 1H), 3.97 (t, *J* = 6.4 Hz, 2H), 3.80 (d, *J* = 14.5 Hz, 1H), 3.31 (m,1H), 3.28–3.24 (m, 1H), 3.21 (dt, *J* = 10.8, 6.4 Hz, 1H), 2.92–2.82 (m, 1H), 2.68–2.60 (m, 1H), 2.46–2.29 (m, 2H), 2.09 (td, *J* = 11.6, 2.7 Hz, 1H), 1.94 (dd, *J* = 14.7, 2.8 Hz, 1H), 1.90–1.73 (m, 3H), and 1.67 (t, *J* = 11.0 Hz, 1H). ^13^C NMR (126 MHz, DMSO-*d*_6_): δ 166.1, 156.4 (d, *J* = 235.6 Hz), 155.0, 137.5, 130.5, 123.8, 120.0, 117.8, 115.7 (d, *J* = 27.7 Hz), 115.6 (d, *J* = 3.8 Hz), 112.2, 66.4, 65.6, 56.3, 54.4, 51.6, 48.7, 41.1, 26.4, and 24.3. HRMS (ESI) *m*/*z* calcd for C_22_H_25_FN_3_O_2_ [M + H]^+^, 382.1925; found: 382.1927.

### Kinetic and Intrinsic Solubility Determination

All experiments were carried out at a controlled temperature of 25.0 ± 0.2 °C. The pH range of titration assays was set between pH 2.0 to pH 12.0. Prior to use, 0.5 M KOH base titrant was standardized by the titration of approximately 15 mg of potassium hydrogen phthalate, in triplicate. 0.5 M HCl titrant was subsequently standardized against the base titrant. The assay media for solubility determination was kept at a constant ionic strength of 0.15 M KCl and under an argon atmosphere. The pH electrode was calibrated daily using the Avdeef–Bucher four-parameter equation.

The sample was titrated at a starting concentration of 2.4 mM, from pH 2.0 to high pH. The sample precipitated from solution at pH 5.6, as detected by a UV-turbidity probe corresponding to a kinetic solubility of 55.5 μg/mL. After precipitation, base and acid titrants were alternately added to drive the sample back and forth across the equilibrium solubility of the neutral species (the intrinsic solubility). Here, the sample would exist in a supersaturated or subsaturated state (i.e., chase equilibrium). The intrinsic solubility was determined from the pH between the supersaturated and subsaturated states corresponding to an aqueous intrinsic solubility of 6.58 μg/mL.

### Human Plasma Protein Binding Determination

Fractional binding of the test article (ITI-333) to human plasma proteins was determined using rapid equilibrium dialysis. 300 μL of human plasma sample containing the test article (ITI-333, 5 μM) was prewarmed and added to the sample chamber of the RED insert, and then 500 μL of PBS was added to the buffer chamber of the insert. The RED base plate was placed on a thermomixer which was prewarmed to 37 °C. Chambers were allowed to reach equilibrium while shaking at 300 rpm for 4 hours. Aliquots were removed from the sample chamber (donor) and from the buffer chamber (receiver) for sample analysis. Samples were diluted to achieve the same analytical matrix and analyzed using LC–MS/MS. For quality control, the assay was run in duplicate (*n* = 2), and reference compound warfarin (10 μM) was codosed with the test article to ensure no leakage of the chambers during the experiment and that the chambers reached equilibrium. Percent binding to human plasma proteins was calculated as follows
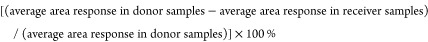


### Analgesic Effect Evaluation in the Tail Flick Studies in CD-1 Mice

All animals were cared for in accordance with the *Guide for the Care and Use of Laboratory Animals* of the Institute of Laboratory Animal Resources, National Research Council, and all procedures were performed with the approval of the Institutional Animal Care and Use Committees at the respective institutions and contract research organizations. Male CD-1 mice (Charles River Laboratories) were examined, handled, and weighed prior to initiation of the study to ensure adequate health and suitability. The mice were maintained on a 12/12 light/dark cycle with lights on at 7:00 am EST. The room temperature was maintained between 20 and 23 °C with a relative humidity maintained around 50%. Chow and water were provided *ad libitum* for the duration of the study. Prior to the start of testing, animals were weighed and balanced across body weight and treatment.

In the tail flick study with acute dosing, morphine (Sigma; Lot# 041M1441 V; 5 mg/kg freebase) was dissolved in saline and administered subcutaneously 30 min prior to testing at a dose volume of 10 mL/kg. Compound 5 (ITI-333) was dissolved in 45% β-cyclodextrin and injected subcutaneously 30 min prior to testing at a dose of 0.003, 0.01, 0.03, 0.1, 0.3, or 1 mg/kg, with a dosing volume of 10 mL/kg.

In the tail flick study examining the analgesic effect attenuated by naloxone, morphine (Sigma; Lot# 041M1441 V; 5 mg/kg freebase) was dissolved in saline and administered subcutaneously 30 min prior to testing at a dose volume of 10 mL/kg. Naloxone (3 mg/kg) was dissolved in saline and administered intraperitoneally 20 min prior to treatment with morphine or ITI-333. ITI-333 was dissolved in 0.25% methylcellulose and administered orally 30 min prior to testing at a dose of 1, 3, or 10 mg/kg, with a dosing volume of 10 mL/kg.

In the tail flick study examining subchronic dosing, vehicle or ITI-333 (0.3 or 3 mg/kg) in 45% beta-cyclodextrin (in water) was administered to mice subcutaneously (s.c.) once daily for 14 days at a dose volume of 10 mL/kg. On day 15, mice were administered ITI-333 (0.01, 0.03, 0.1, 0.3, 1, or 3 mg/kg) or vehicle subcutaneously 30 min prior to testing at a dose volume of 10 mL/kg.

The tail flick (TF) test measures the pain reflex threshold in restrained animals, a measure of acute nociception organized at the level of the spinal cord. Animals were placed inside the holding enclosure with the tails stretched out and placed over a small window on the TF platform that housed an infrared heat source. A foot switch was used to activate the light beam source directed at the tail (IR intensity 75). When the pain threshold was reached, the mouse flicked the tail away from the heat source, and the latency to respond was recorded automatically by the TF apparatus. The mouse could withdraw the tail at any point when the temperature of the tail became too uncomfortable. Mice were administered test treatment according to the study design. Following the pretreatment time, mice were tested on the TF apparatus. Three consecutive trials were recorded per animal with a 10 s trial cut off. At the end of the experiment, the mice were euthanized. The TF apparatus was cleaned between each animal. Data were analyzed by analysis of variance (ANOVA) followed by posthoc comparisons with Fisher tests when appropriate. An effect was considered significant if *p* < 0.05. Statistical outliers that fell above or below 2 standard deviations from the mean were removed from the final analysis. To determine the ITI-333 dose corresponding to 50% of the maximum possible effect (MPE), or ED_50_, for the acute tail flick assay, normalized dose–response data were analyzed in GraphPad Prism 10.1.1 using nonlinear regression with a Hill slope of 1.0 and top and bottom constraints of 0 and 100%, respectively. Response latency data were normalized by calculating the % MPE using the following formula: % MPE = [(test latency – control latency)/(maximum latency – control latency)] × 100, where “test latency” was the response latency measured for each mouse, “control latency” was the mean response latency for the vehicle control group, and “maximum latency” was the cutoff latency (10 s).

### Analgesic Effect Evaluation in Inflammatory Pain Using the Mouse Formalin Test

All animals were cared for in accordance with the *Guide for the Care and Use of Laboratory Animals* of the Institute of Laboratory Animal Resources, National Research Council, and all procedures were performed with the approval of the Institutional Animal Care and Use Committees at the respective institutions and contract research organizations. Male C57Bl/6 mice (Jackson Laboratories; 7 weeks of age upon arrival) were used in the study. All mice were examined, handled, and weighed prior to initiation of the study to ensure adequate health and suitability. The mice were maintained on a 12/12 light/dark cycle with lights on at 7:00 am EST. The room temperature was maintained between 20 and 23 °C with a relative humidity maintained around 50%. Chow and water were provided *ad libitum* for the duration of the study. Prior to the start of testing, animals were weighed and balanced across body weight and treatment.

Analgesic effects of compound **5** (ITI-333) were studied in mice against inflammatory pain induced by the administration of a chemical irritant (formalin). On the day of the experiment, mice were habituated in an observation chamber. Animals received an injection of vehicle delivered either SC (saline) or PO (45% cyclodextrin in water), an SC injection of morphine (5 mg/kg in saline) or compound **5** (ITI-333; 0.3, 1, or 3 mg/kg in 45% cyclodextrin in water), or a PO injection of compound **5** (ITI-333; 3 mg/kg in 45% cyclodextrin in water). Thirty min later, all mice received a single SC injection of formalin (2.5% solution in water) into the plantar surface of the left hind paw. Mice were returned to the observation chamber and immediately observed for licking behavior. Formalin injection produced a biphasic response in mice: an acute, nociception first response and a second, tonic, late-phase inflammatory response. The total time spent by mice licking or biting the treated hind paw over the next 40 min was recorded. The acute response was evaluated for 0–6 min, and the late phase response was recorded from 7–40 min. Observations were made continuously. The total time spent licking was then calculated for each mouse and averaged across the group. All test animals were euthanized at the end of the study. Data were analyzed by analysis of variance (ANOVA) followed by posthoc comparisons with Fisher tests when appropriate. An effect was considered significant if *p* < 0.05. Data are represented as the mean and standard error to the mean. Statistical outliers that fell above or below 2 standard deviations from the mean were removed from the final analysis.

## Supporting Information Available

Procedures for receptor binding assays, functional assays and pharmacokinetic studies, and representative HPLC chromatograms of the final target compounds (PDF)
Formula strings for all final target compounds (CSV)

## Abbreviations

CHO cells: Chinese hamster ovary cells
CNS: central nervous system
DAMGO: [d-Ala2, MePhe4, Gly-ol5]-enkephalin
^125^DOI: (±)-1-(2,5-dimethoxy-4-[^125^I]-iodophenyl)-2-aminopropane
D_1_R: dopamine D_1_ receptors
DBU: 1,8-diazabicyclo[5.4.0]undec-7-ene
DIPEA: *N*,*N*-diisopropylethylamine
DMAc: dimethylacetamide
DMAP: 4-dimethylaminopyridine
DMF: dimethylformamide
ESI: electrospray ionization
GPCR: G protein coupled receptor
HEK-293: a cell line exhibiting epithelial morphology that was isolated from the kidney of a human embryo
[^3^H]-7-OH-DPAT: [^3^H]7-hydroxy-*N*,*N*-di-*n*-propyl-2-aminotetralin
HRMS: high-resolution mass spectrometry
5-HT: serotonin or 5-hydroxytryptamine
[^3^H]SCH 23390: *R*-(+)-8-chloro-5-phenyl-3-(tritritiomethyl)-2,3,4,5-tetrahydro-1*H*-benzo[*d*]azepin-7-ol
LLE: ligand–lipophilicity efficiency
MOR: μ-opioid receptor
PO: oral administration
SAR: structure–activity relationship
SC: subcutaneous administration
SEM: standard error of the mean
SERT: serotonin transporter
