# Supplementary material for: Discovery of ITI-333, a Novel Orally Bioavailable Molecule Targeting Multiple Receptors for the Treatment of Pain and Other Disorders
Source: J Med Chem. 2024 May 28;67(11):9355–73. doi: 10.1021/acs.jmedchem.4c00480 (PMC11181336; doi:10.1021/acs.jmedchem.4c00480)
Supplement: Supplementary file 1 — jm4c00480_si_001.pdf [file jm4c00480_si_001.pdf]

Supporting Information

Discovery of ITI-333, a Novel Orally Bioavailable Molecule Targeting Multiple Receptors for the Treatment of Pain and Other Disorders

Peng Li,[*,†] Qiang Zhang,[†] Hailin Zheng,[†] Yupu Qiao,[†] Gretchen L. Snyder,[†] Terry Martin,[†] Wei Yao,[†] Lei Zhang[†] and Robert E. Davis [†]

[†]Intra-Cellular Therapies, Inc., 430 East 29th Street, Suite 900, New York, New York, 10016, United States

***Corresponding author:**

Peng Li, Ph.D.

Intra-Cellular Therapies, Inc.

430 East 29th Street, Suite 900

New York, NY 10016

Phone: 646-440-9388

Email: pli@itci-inc.com

Table of Contents

Representative HPLC Chromatograms of the Final Compounds S3—S5

In Vitro Binding Assays S6—S9

In Vitro Functional Assays S10

Pharmacokinetic Study of Compound 5 (ITI-333) After Intravenous and Oral Administration to Cynomolgus Monkeys S11

References S12

Representative HPLC Chromatograms of the Final Compounds

The representative HPLC chromatograms were amplified to show the retention times of the final compounds and purity data clearly.

Compound 5

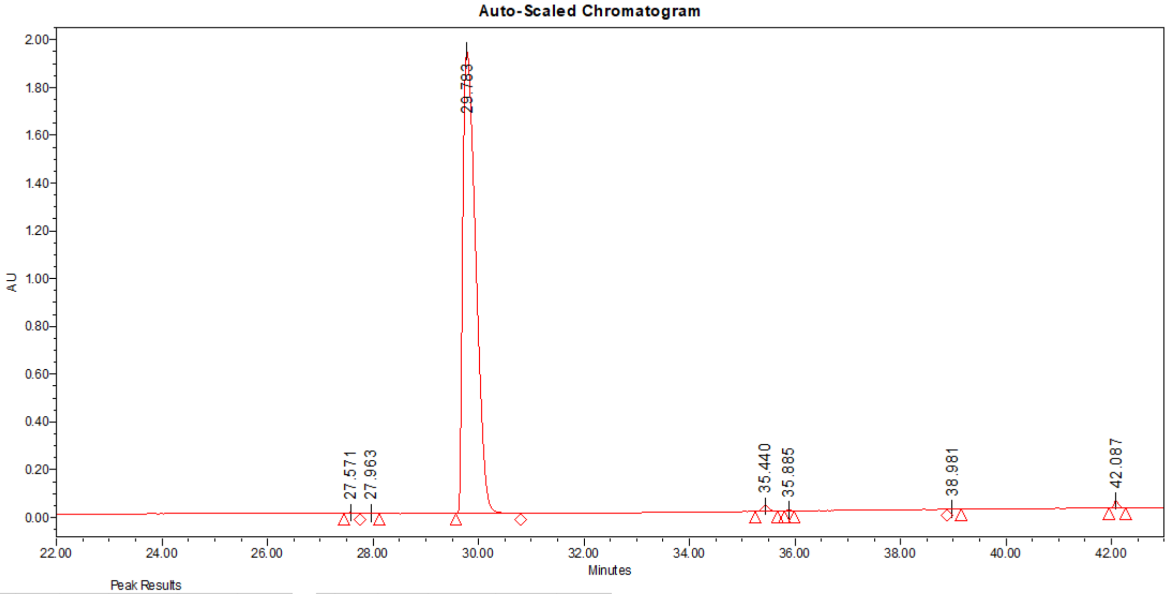

Peak Results

	Name	RT	Area	Height	% Area
1		27.571	34257	4086	0.10
2		27.963	31487	3476	0.09
3		29.783	32987699	1936674	98.49
4		35.440	211783	23177	0.63
5		35.885	22597	4722	0.07
6		38.981	21041	3606	0.06
7		42.087	184178	30494	0.55

Compound 14

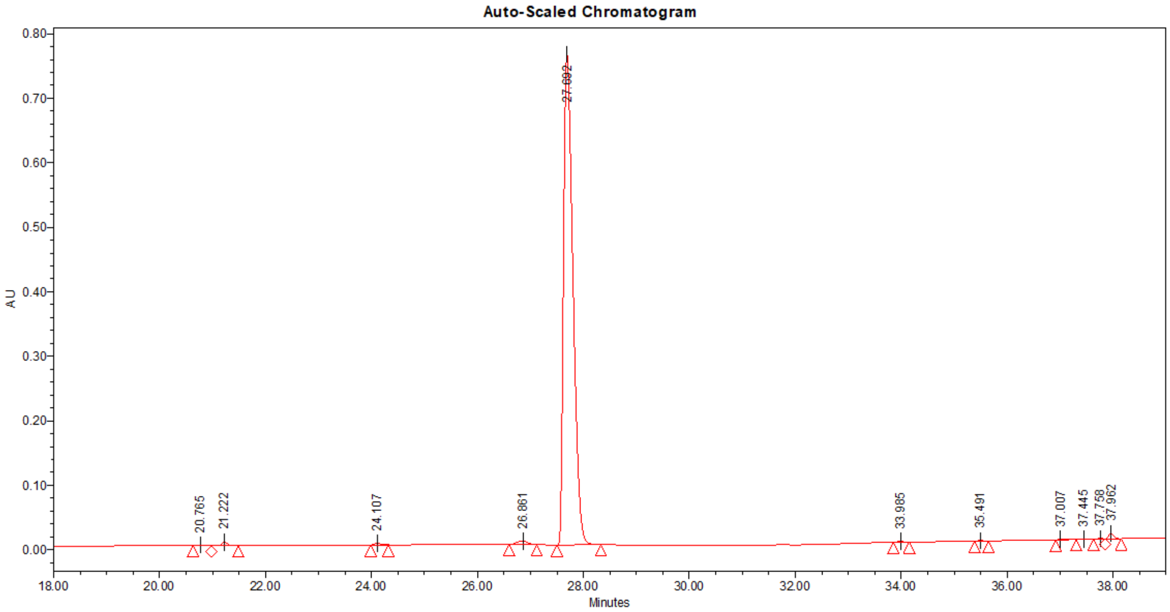

Peak Results

	Name	RT	Area	Height	% Area
1		20.765	10663	1259	0.11
2		21.222	43763	6097	0.45
3		24.107	24602	3132	0.25
4		26.861	80687	5481	0.84

	Name	RT	Area	Height	% Area
5		27.692	9396279	766855	97.37
6		33.985	9622	1274	0.10
7		35.491	5521	828	0.06
8		37.007	6413	734	0.07

	Name	RT	Area	Height	% Area
9		37.445	6764	1009	0.07
10		37.758	8551	1353	0.09
11		37.962	56869	8866	0.59

Compound 20

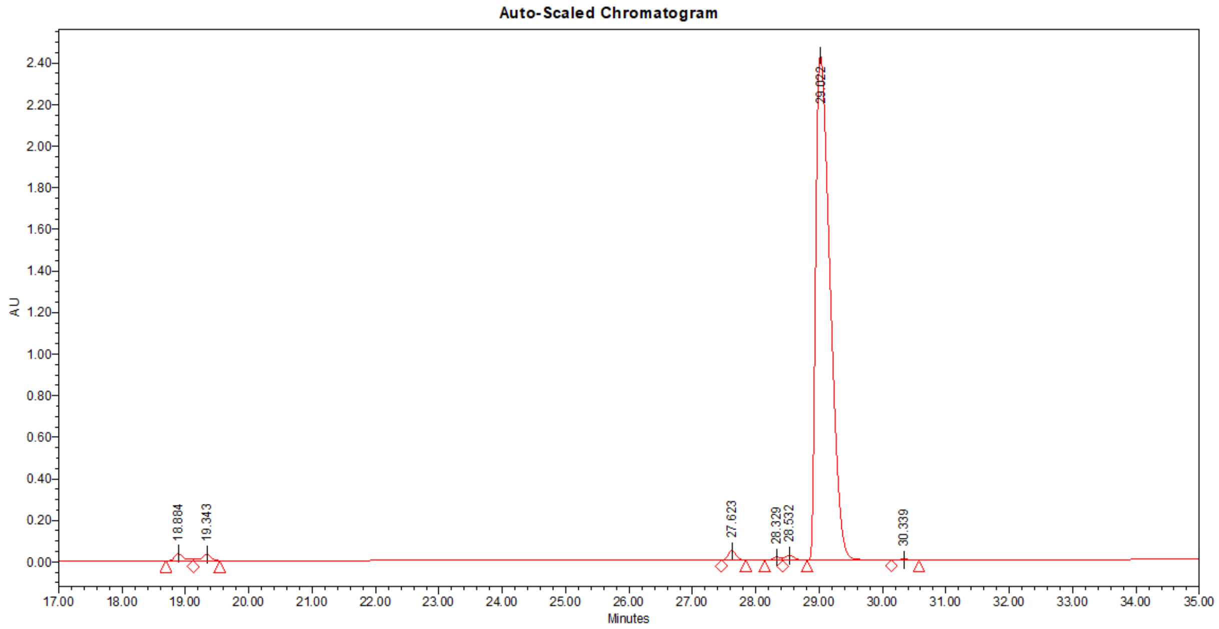

Peak Results

	Name	RT	Area	Height	% Area
1		18.884	331384	34547	0.83
2		19.343	304344	31927	0.76
3		27.623	392019	45293	0.98
4		28.329	138628	15899	0.35

	Name	RT	Area	Height	% Area
5		28.532	247028	25073	0.62
6		29.022	38354679	2430941	96.31
7		30.339	58148	4985	0.15

In Vitro Binding Assays

Compounds were dissolved at 10 mM in DMSO fresh each day and serially diluted in binding buffer leaving no more than 1% DMSO, a concentration without influence on binding. Generally, eight concentrations of inhibitor were tested in duplicate and the IC_{50} inhibition values were corrected to K_i affinity values using the Cheng-Prusoff relationship.[1] The reported K_i or percentage of inhibition values are the means of at least two determinations.

5-HT$_{2A}$ receptor radioligand binding assay. The binding affinity of synthesized compounds against serotonin 5-HT_{2A} receptors was determined by a radioligand displacement method.[2] [^{125}I]DOI was used as a radioligand. The assay was conducted by Eurofins Cerep (assay ID: 0471; link: https://www.eurofinsdiscoveryservices.com/catalogmanagement/viewItem/5-HT2A-Human-Serotonin-GPCR-Binding-Agonist-Radioligand-Assay-Cerep/471). In this assay, cell membrane homogenates (30 µg protein) were incubated for 60 min at 22° C with 0.1 nM [^{125}I]DOI in the absence or presence of the test compound in a buffer containing 50 mM Tris-HCl (pH 7.4), 5 mM $MgCl_2$, 10 µM pargyline and 0.1% ascorbic acid. Nonspecific binding was determined in the presence of 1 µM DOI. Following incubation, the samples were filtered rapidly under vacuum through glass fiber filters (GF/B, Packard) presoaked with 0.3% PEI and rinsed several times with ice-cold 50 mM Tris-HCl using a 96-sample cell harvester (Unifilter, Packard). The filters were dried, then counted for radioactivity in a scintillation counter (Topcount, Packard) using a scintillation cocktail (Microscint 0, Packard). The results were expressed as a percent inhibition of the control radioligand specific binding. The standard reference compound is DOI, which was tested in each experiment at several concentrations to obtain a competition curve from which its IC_{50} was calculated.

Dopamine D$_2$ receptor binding assay. The binding affinity of synthesized compounds against dopamine D_2 receptors was determined by a radioligand displacement method.[3] [^{3}H]7-OH-DPAT was used as a radioligand. The assay was conducted by Eurofins Cerep (assay ID: 1322; link: https://www.eurofinsdiscoveryservices.com/catalogmanagement/viewItem/D2S-Human-Dopamine-GPCR-Binding-Agonist-Radioligand-Assay-Cerep/1322). In this assay, cell membrane homogenates (24 µg protein) were incubated for 60 min at 22 °C with 1 nM [^{3}H]7-OH-DPAT in the absence or presence of the test compound in a buffer containing 50 mM Tris-HCl (pH 7.4), 5 mM KCl, 5 mM $MgCl_2$, 1 mM EDTA and 0.1% BSA. Nonspecific binding was determined in the presence of 10 µM (+)butaclamol. Following incubation, the samples were fil-

tered rapidly under vacuum through glass fiber filters (GF/B, Packard) presoaked with 0.3% PEI and rinsed several times with ice-cold 50 mM Tris-HCl using a 96-sample cell harvester (Unifilter, Packard). The filters were dried, then counted for radioactivity in a scintillation counter (Topcount, Packard) using a scintillation cocktail (Microscint 0, Packard). The results were expressed as a percent inhibition of the control radioligand specific binding. The standard reference compound is 7-OH-DPAT, which was tested in each experiment at several concentrations to obtain a competition curve from which its IC_{50} was calculated.

Dopamine D_1 receptor binding assay. The binding affinity of synthesized compounds against dopamine D_1 receptors was determined by a radioligand displacement method.[4] [^{3}H]SCH 23390 was used as a radioligand. The assay was conducted by Eurofins Cerep (assay ID: 0044; link: https://www.eurofinsdiscoveryservices.com/catalogmanagement/viewItem/D1-Human-Dopamine-GPCR-Binding-Antagonist-Radioligand-Assay-Cerep/44). In this assay, Cell membrane homogenates (60 µg protein) were incubated for 60 min at 22 °C with 0.3 nM [^{3}H]SCH 23390 in the absence or presence of the test compound in a buffer containing 50 mM Tris-HCl (pH 7.4), 5 mM KCl, 5 mM $MgCl_2$, 1.5 mM $CaCl_2$ and 5 mM EDTA. Nonspecific binding was determined in the presence of 1 µM SCH 23390. Following incubation, the samples were filtered rapidly under vacuum through glass fiber filters (GF/B, Packard) presoaked with 0.3% PEI and rinsed several times with ice-cold 50 mM Tris-HCl using a 96-sample cell harvester (Unifilter, Packard). The filters were dried then counted for radioactivity in a scintillation counter (Topcount, Packard) using a scintillation cocktail (Microscint 0, Packard). The results were expressed as a percent inhibition of the control radioligand specific binding. The standard reference compound is SCH 23390, which was tested in each experiment at several concentrations to obtain a competition curve from which its IC_{50} was calculated.

µ-Opioid receptor binding assay. The binding affinity of synthesized compounds against µ-opioid receptors was determined by a radioligand displacement method.[5] [^{3}H]DAMGO was used as a radioligand. The assay was conducted by Eurofins Cerep (assay ID: 0118; link: https://www.eurofinsdiscoveryservices.com/catalogmanagement/viewItem/mu-MOP-Human-Opioid-GPCR-Binding-Agonist-Radioligand-Assay-Cerep/118). In this assay, cell membrane homogenates (60 µg protein) were incubated for 120 min at 22 °C with 0.5 nM [^{3}H]DAMGO in the absence or presence of the test compound in a buffer containing 50 mM Tris-HCl (pH 7.4) and 5 mM $MgCl_2$. Nonspecific binding was determined in the presence of 10 µM naloxone. Fol-

lowing incubation, the samples were filtered rapidly under vacuum through glass fiber filters (GF/B, Packard) presoaked with 0.3% PEI and rinsed several times with ice-cold 50 mM Tris-HCl using a 96-sample cell harvester (Unifilter, Packard). The filters were dried, then counted for radioactivity in a scintillation counter (Topcount, Packard) using a scintillation cocktail (Microscint 0, Packard). The results were expressed as a percent inhibition of the control radioligand specific binding. The standard reference compound is DAMGO, which was tested in each experiment at several concentrations to obtain a competition curve from which its IC_{50} was calculated.

Serotonin transporter binding assay. The binding affinity of synthesized compounds against serotonin transporters was determined by a radioligand displacement method.[6] [^{3}H]imipramine was used as a radioligand. The assay was conducted by Eurofins Cerep (assay ID: 0439; link: https://www.eurofinsdiscoveryservices.com/catalogmanagement/viewItem/SET-Human-Serotonin-Transporter-Binding-Antagonist-Radioligand-Assay-Cerep/439). In this assay, cell membrane homogenates (12 µg protein) were incubated for 60 min at 22 °C with 2 nM [^{3}H]imipramine in the absence or presence of the test compound in a buffer containing 50 mM Tris-HCl (pH 7.4), 120 mM NaCl, 5 mM KCl and 0.1% BSA. Nonspecific binding was determined in the presence of 10 µM imipramine. Following incubation, the samples were filtered rapidly under vacuum through glass fiber filters (GF/B, Packard) presoaked with 0.3% PEI and rinsed several times with an ice-cold buffer containing 50 mM Tris-HCl and 150 mM NaCl using a 96-sample cell harvester (Unifilter, Packard). The filters were dried, then counted for radioactivity in a scintillation counter (Topcount, Packard) using a scintillation cocktail (Microscint 0, Packard). The results were expressed as a percent inhibition of the control radioligand specific binding. The standard reference compound is imipramine, which was tested in each experiment at several concentrations to obtain a competition curve from which its IC_{50} was calculated.

Adrenergic α_{1A} receptor binding assay. The binding affinity of synthesized compounds against adrenergic α_{1A} receptors was determined by a radioligand displacement method.[7] [^{3}H]prazosin was used as a radioligand. The assay was conducted by Eurofins Cerep (assay ID: 2338; link: https://www.eurofinsdiscoveryservices.com/catalogmanagement/viewItem/alpha1A-Human-Adrenoceptor-GPCR-Binding-Antagonist-Radioligand-Assay-Cerep/2338). In this assay, cell membrane homogenates (20 µg protein) were incubated for 60 min at 22 °C with 0.1 nM [^{3}H]prazosin in the absence or presence of the test compound in a buffer containing 50 mM Tris-HCl (pH 7.4), 0.5 mM EDTA, 20 mg/l aprotinin and 0.01% bacitracin. Nonspecific binding was

determined in the presence of 0.1 mM epinephrine. Following incubation, the samples were filtered rapidly under vacuum through glass fiber filters (GF/B, Packard) presoaked with 0.3% PEI and rinsed several times with ice-cold 50 mM Tris-HCl using a 96-sample cell harvester (Unifilter, Packard). The filters were dried, then counted for radioactivity in a scintillation counter (Topcount, Packard) using a scintillation cocktail (Microscint 0, Packard). The results were expressed as a percent inhibition of the control radioligand specific binding. The standard reference compound is WB 4101, which was tested in each experiment at several concentrations to obtain a competition curve from which its IC_{50} was calculated.

All cell lines listed above were tested mycoplasma free. More information about these cell lines is listed in Table S1.

Table S1. Information on the Cell Lines used in Receptor Binding Assays

Item	Assays	Cell	Origin of the cell line	Date received/created	Cell type	Class	Containment level	OGM	Accession number
471	$5\text{-}HT_{2A}$ (h)	HEK 293 / $5\text{-}HT_{2A}$ (h)	Cerep Rueil-Malmaison (Molecular biology service)	30/06/2002	Human Embryonic Kidney (HEK 293) expressing the $5\text{-}HT_{2A}$ human receptor	1	2	yes	BC074849 P28233
44	D_1 (h)	CHO / D_1 (h)	Inserm	14/10/2003	Chinese Hamster Ovary (CHO) expressing the D_1 human receptor	1	1	yes	P21728
118	Mu (h) (MOP)	HEK 293 / Mu (h)	Inserm	09/10/2003	Human Embryonic Kidney (HEK 293) expressing the Mu human receptor	1	2	yes	BC074927 P35372
439	5-HT transporter (h)	CHO-S / SERT (h)	Cerep Rueil-Malmaison (Molecular biology service)	17/03/2005	Chinese Hamster Ovary (CHO) expressing the 5-HT transporter human receptor	1	1	yes	NM_001045 P31645
1322	D_{2S} (h)	HEK 293 / D_{2S} (h)	University of Oregon (USA)	16/09/2004	Human Embryonic Kidney (HEK 293) expressing the D_{2S} human receptor	1	2	yes	NM_016574.2 P14416

In Vitro Functional Assays

5-HT$_{2A}$ receptors. ITI-333 functional activity was evaluated in a whole cell-based assay system in which 5-HT$_{2A}$ receptor-dependent calcium signaling was measured using a recombinant calcium-dependent bioluminescent protein. ITI-333 was studied in the agonist mode and for its ability to antagonize the activity of the 5 HT$_{2A}$ receptor full agonist α-methylserotonin.

μ-Opioid receptors. ITI-333 functional activity was examined in CHO cells expressing human recombinant μ-opioid receptors; agonist activity (using 1 μM DAMGO as a control) and antagonist activity (using reversal of DAMGO-inhibited cAMP production) were assessed at drug concentrations between 0.056 nM–10 μM. Buprenorphine and naloxone (0.0056 nM–1 μM) were used as comparators for agonist and antagonist activity, respectively. The functional activity of ITI-333 at μ-opioid receptors was further investigated in a whole cell-based assay using μ-opioid receptor-dependent suppression of adenylyl cyclase activity to determine intrinsic efficacy compared with DAMGO. The effect of ITI-333 on β-arrestin pathway signaling at μ-opioid receptors was also investigated using the PathHunter® β-Arrestin assay (DiscoverX, Fremont, CA) using met-enkephalin as a control.

Dopamine D$_1$ receptors. ITI-333 functional activity at dopamine D$_1$ receptors was determined at a single concentration of 10 μM. Cellular agonist effects were calculated as the percent of control response to 10 μM dopamine; antagonist effects were calculated as the percent inhibition of 300 nM dopamine response in CHO cells expressing human recombinant D$_1$ receptors.

Adrenergic α$_{1A}$ receptors. ITI-333 functional activity on adrenergic α$_{1A}$ receptor-dependent calcium signaling was assessed using a whole cell-based AequoZen assay system. Adrenergic α$_{1A}$ receptor activation was detected via measurement of light emission after addition of increasing concentrations of ITI-333 or the positive control agonist phenylephrine (≤ 100 μM) in CHO-K1 cells with stable co-expression of aequorin and adrenergic α$_{1A}$ receptors. In antagonist mode, the reversal of agonist-induced (50 nM phenylephrine) calcium response by increasing concentrations of ITI 333 (≤10 μM) or a positive control (tamsulosin [≤1 μM]) was examined.

Pharmacokinetic (PK) Study of Compound 5 (ITI-333) After Intravenous and Oral Administration to Cynomolgus Monkeys.

Compound **5** (ITI-333) was administered to fasted groups of non-naïve male Cynomolgus monkeys by either intravenous (IV) injection (n=3) or oral gavage (n=4). The dose solution for IV was 5 mg/mL of ITI-333 free base in 45% Trappsol with 1% DMSO in water. The dose solution for PO was 2 mg/mL of ITI-333 free base in PEG 400. Blood samples were collected pre-dose and at 0.033, 0.083, 0.25, 0.5, 1, 2, 4, 6, 8, 24, 36 and 48 h after IV injection. Blood samples were collected at nominal timepoints, 0.083, 0.5, 2, 6, 12, 24 and 48 h following PO administration. All PK blood samples were processed to plasma (200 µL) and were stabilized with 20 µL of 20 mg/mL ascorbic acid solution. Concentrations of ITI-333 free base were analyzed for all samples using a liquid chromatography tandem mass spectrometry method. PK parameters, such as area under the concentration-time curve (AUC), were based on the group mean concentration versus time data, and were calculated using non-compartmental pharmaceutical data analysis software PK Solutions 2.0 (Summit Research Services, CO). Oral bioavailability was calculated as:

$$F\% = \text{Dose-normalized } AUC_{PO} / \text{Dose-normalized } AUC_{IV}$$

References

1) Cheng, Y.; Prusoff, W. H. Relationship between the inhibition constant (K_i) and the concentration of inhibitor which causes 50 per cent inhibition (IC_{50}) of an enzymatic reaction. *Biochem. Pharmacol.* **1973**, *22*, 3099-3108.

2) Bryant, H. U.; Nelson, D. L.; Button, D.; Cole, H. W.; Baez, M. B.; Lucaites, V. L.; Wainscott, D. B.; Whitesitt, C.; Reel, J.; Simon, R.; Koppel, G. A. A novel class of 5-HT_{2A} receptor antagonist: aryl aminoguanidines. *Life Sci.*, **1996**, *15*, 1259-1268.

3) Grandy, D. K.; Marchionni, M. A.; Makam, H.; Stofko, R. E.; Alfano, M.; Frothingham, L.; Fischer, J. B.; Burke-Howie, K. J.; Bunzow, J. R.; Server, A. C; Civelli, O. Cloning of the cDNA and gene for a human D_2 dopamine receptor. *Proc. Natl. Acad. Sci. USA*, **1989**, *86*, 9762-9766.

4) Zhou, Q.-Y.; Grandy, D. K.; Thambi, L.; Kushner, J. A.; Van Tol. H. H. M.; Cone, R.; Pribnow W, D.; Salon, J.; Bunzow, J. R.; Civelli, O. Cloning and expression of human and rat D_1 dopamine receptors. *Nature*, **1990**, *347*, 76-80.

5) Wang, J.-B.; Johnson, P. S.; Persico, A. M.; Hawkins, A. L.; Griffin, C. A.; Uhl, G. R. Human μ-opiate receptor. cDNA and genomic clones, pharmacological characterization and chromosomal assignment. *FEBS Lett.*, **1994**, *338*, 217-222.

6) Tatsumi, M.; Jansen, K.; Blakely, R. D.; Richelson, E. Pharmacological profile of neuroleptics at human monoamine transporters. *Eur. J. Pharmacol.*, **1999**, *368*, 277-283.

7) Schwinn, D. A.; Lomasney, J. W.; Lorenz, W.; Szklut, P. J.; Fremeau, R. T. Jr; Yang,-Feng, T.L., Caron, M. G.; Lefkowitz, R. J.; Cotecchia, S. Molecular cloning and expression of the cDNA for a novel alpha1-adrenergic receptor subtype. *J. Biol. Chem.*, **1990**, *265*, 8183-8189.
